# Gut Microbiota‐Derived Anandamide Mediates the Therapeutic Effects of Urolithin A on Alcohol‐Induced Cognitive and Social Dysfunction via CB1R‐DRD2‐RAP1 Signaling Axis

**DOI:** 10.1002/advs.202508048

**Published:** 2026-02-03

**Authors:** Hongbo Zhang, Zibin Li, Yao Xiao, Ji Bian, Caian He, Chao Liu, Lan Gong, Lin Han, Zhigang Liu, Min Wang

**Affiliations:** ^1^ Department of Nutrition and Health College of Food Science and Engineering Northwest A&F University Yangling Shaanxi China; ^2^ College of Food Science and Engineering Ningxia University Yinchuan Ningxia China; ^3^ Kolling Institute Sydney Medical School Royal North Shore Hospital University of Sydney St. Leonards NSW Australia; ^4^ Key Laboratory of Novel Food Resources Processing Ministry of Agriculture and Rural Affairs/Institute of Agro‐Food Science and Technology Shandong Academy of Agricultural Sciences Jinan P. R. China; ^5^ UNSW Microbiome Research Centre St George and Sutherland Clinical Campus University of New South Wales Sydney NSW Australia

**Keywords:** alcohol‐induced cognitive and social dysfunction, anandamide, *Bacteroids sartorii* and *Parabacteroids distasonis*, dopamine D2 receptor and cannabinoid receptor 1, urolithin A

## Abstract

Chronic alcohol consumption disrupts the gut microbiome, exacerbating alcohol‐induced cognitive and social dysfunction (AICSD), which constitutes a primary etiology of early‐onset dementia. Urolithin A (UA) has been well‐reported as an effective intervention for neurodegenerative diseases. However, the protective efficacy of UA against AICSD, and its underlying mechanisms remain largely elusive. First, our study demonstrates that UA significantly enhances work memory (60.43%), short‐term memory (12‐fold), long‐term memory (50.32%), social ability (10‐fold), and social novelty (12‐fold), while concurrently reducing synaptic impairments and neuroinflammation. Moreover, UA restores AICSD by upregulating the dopamine D2 receptor (DRD2) via RAP1 signaling. Furthermore, antibiotic treatment and fecal microbiota transplantation experiments confirm the causality between the host microbiota and behavioral alterations. Treatment with UA‐enriched *Bacteroids sartorii* and *Parabacteroids distasonis*, or their derived endocannabinoid‐anandamide (AEA), also ameliorates AICSD. Finally, AEA inhibits the Rap1 signaling through cannabinoid receptor 1 (CB1R) and DRD2 interaction, eventually ameliorating AICSD. Collectively, our study elucidates that microbiota‐derived AEA mediates the therapeutic effects of UA on AICSD through the CB1R‐DRD2‐RAP1 signaling axis, providing valuable insights for UA and microbiome‐targeted endocannabinoid interventions against AICSD.

## Graphical abstract

This study reveals that Urolithin A (UA) counteracts alcohol‐induced cognitive and social dysfunction (AICSD) via a gut microbiome‐dependent mechanism. UA‐enriched Bacteroids sartorii and Parabacteroids distasonis elevate anandamide (AEA), which activates the CB1R‐DRD2‐Rap1 signaling cascade to drive synaptic repair and reduce neuroinflammation, thereby elucidating a novel gut‐to‐brain communication

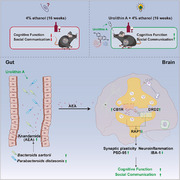

## 1 Introduction

Chronic alcohol consumption ranks as the third most significant global health risk, with alcohol‐related diseases posing a formidable challenge to public health worldwide [1]. A cohort study has revealed that heavy alcohol consumption is the most significant risk factor for dementia, particularly early‐onset dementia [2]. Even a moderate level of alcohol consumption is associated with detrimental brain outcomes, including hippocampal atrophy, brain aging, and the of entire brain volume reduction [3, 4, 5]. However, progress in the management of alcohol‐induced cognitive and social dysfunction (AICSD) remains limited, underscoring the urgent need to develop innovative and pragmatic therapeutic strategies. Accumulating evidence indicates that chronic alcohol consumption induces neuroinflammation in the hippocampal region of the central nervous system (CNS), resulting in morphological and functional abnormalities in the hippocampal neurons and synapses [6], cognitive impairments, motor deficits, and anxiety‐like behaviors [7, 8, 9]. Moreover, chronic alcohol exposure induces significant dysbiosis of the intestinal microbiota, leading to compromised mucosal barrier function and disrupted gut homeostasis. Recent research has demonstrated that gut microbiota possess the capability to communicate with both the peripheral nervous system and CNS of their host via the gut–brain axis, thereby leading to modifications in neurotransmitter function, brain signaling pathways, and behavioral patterns [10, 11]. Cohort studies have revealed significant interconnections between specific gut microbial profiles, cognitive dysfunction, and hippocampal atrophy, suggesting their pivotal role in the pathogenesis of dementia [12]. Emerging evidence highlights the microbiome–gut–brain axis as a critical modulator of social cognitive function and alcohol craving behaviors in adolescent binge drinkers [13]. Notably, butyrate produced through microbial metabolism demonstrates neuroprotective efficacy against chronic ethanol‐induced central nervous system injury, achieved via dual mechanisms of microglial inflammatory response suppression and bidirectional axis communication reinforcement [14]. Therefore, regulating intestinal microbiota homeostasis and its associated metabolites emerges as a promising strategy for the prevention and treatment of AICSD.

This strategy is strongly supported by a growing body of research on nutritional interventions, particularly regarding functional food components. Numerous studies have consistently demonstrated that intervention with functional food components can effectively enhance brain function by regulating intestinal microbiota [15]. Urolithin A (UA) is derived from ellagic acid and exhibits superior anti‐inflammatory and antioxidant properties. Its biological safety has been extensively validated in human studies [16]. Recently, growing evidence highlights UA as a promising therapeutic agent for preventing neurodegenerative disorders, particularly Alzheimer's disease and Parkinson's disease [17, 18]. Beyond its neuroprotective potential, UA exhibits dual protective effects on intestinal health by reinforcing gut barrier integrity and exerting anti‐inflammatory effects [19].

Notably, emerging research highlights gut microbiota modulation as a central mechanism underlying the therapeutic benefits of UA [20, 21, 22]. Preclinical investigations demonstrate that UA administration effectively mitigates chronic inflammation through gut microbiota regulation, effectively reducing colonic tissue damage in diet‐induced obese rats [20]. In murine colitis models, UA ameliorates intestinal inflammation by restoring microbial balance, modulating tryptophan metabolic pathways, and activating aryl hydrocarbon receptor signaling [21]. Specifically, UA's influence on the murine gut microbiota involves an increase in the abundance of beneficial bacteria, including *Akkermansia*, *Caulobacter crescentus*, *Alloprevotella*, and *Verrucomimicrobiae*, while simultaneously decreasing pathogenic bacteria such as *Colidextribacter*, *Alistipes*, and *Ligilactobacillus*, ultimately leading to an anti‐tumor effect [23]. Moreover, elevated levels of *Lactobacillus* and *Muribaculaceae*, concurrently with diminished levels of *Clostridia_UCG014*, *Candidatus_Saccharimonas*, are posited as potential mechanisms contributing to the muscle‐protective effects of UA [24]. Furthermore, clinically relevant observations suggest UA's diagnostic potential as a biomarker for gut dysbiosis severity and disease progression in Parkinson's patients [22].

Although the neuroprotective and intestinal protective properties of UA have been well‐recognized, its ameliorative effect on AICSD and the underlying mechanisms remain elusive. In light of this, we undertook a comprehensive study to investigate the impact of UA on AICSD and the mediating mechanism of intestinal microbiota in its therapeutic efficacy. We find that UA significantly ameliorates AICSD by activating the DRD2 target in the hippocampus. *Bacteroids sartorii* (*B. sartorii*) and *Parabacteroids distasonis* (*P. distasonis*) and their‐derived endocannabinoid AEA mediate the therapeutic effects of UA on AICSD. AEA enhances the interaction of cannabinoid receptor 1 (CB1R) and dopamine D2 receptor (DRD2) to further ameliorate AICSD through RAP1 signaling.

## 2 Results

### 2.1 UA Ameliorates Alcohol‐Induced Cognitive and Social Dysfunction

**FIGURE 1:**
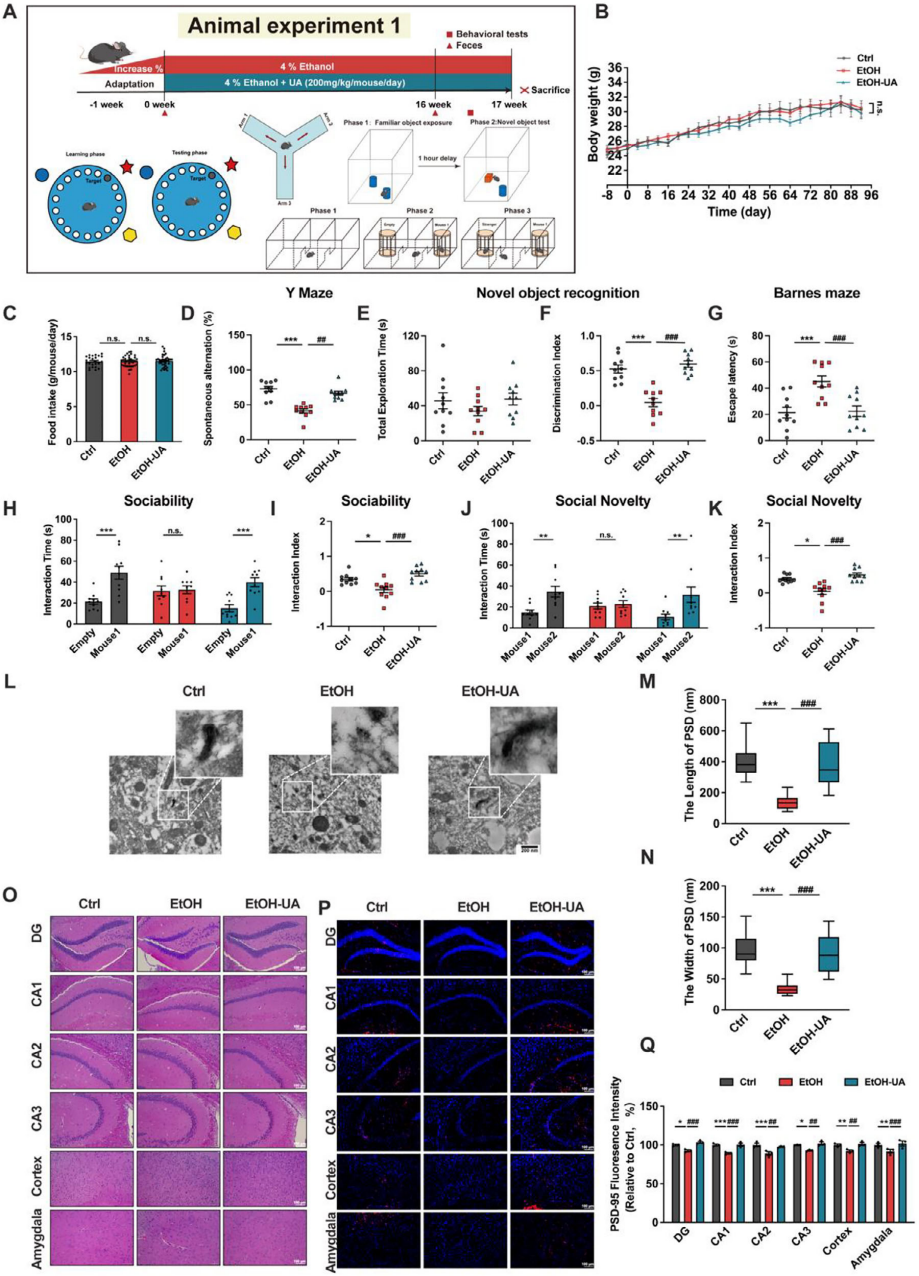
UA ameliorates alcohol‐induced cognitive, social, and synaptic impairments. (A) Timeline of animal experiment 1 depicting the alcohol liquid diet with UA treatment. (B) Body weight (*n* = 10 mice per group). (C) Food intake. (D) For the Y‐maze, spontaneous alternations were recorded (*n* = 10 mice per group). (E,F) For the novel object recognition test, the discrimination index between the novel and familiar objects was calculated (*n* = 10 mice per group). (G) For the Barnes maze, escape latency was recorded (*n* = 10 mice per group). (H) In the sociability test, the time spent interacting with a mouse or with an empty wire cage was recorded (*n* = 10 mice per group). (I) In the sociability test, the interaction index was calculated (*n* = 10 mice per group). (J) In the social novelty test, the time spent interacting with a novel versus a familiar mouse was recorded (*n* = 10 mice per group). (K) In the social novelty test, the interaction index was calculated (*n* = 10 mice per group). (L) Representative images of synapse ultrastructure. (M) The length of PSD (*n* = 3 slices per group). (N) The width of PSD (*n* = 3 slices per group). (O) Representative images of H&E staining of the brain (*n* = 3 mice per group, scale bars, 100 µm). (P,Q) Representative images of immunofluorescence images of PSD‐95 in the brain (*n* = 3 mice per group, scale bars, 100 µm). Data presented as mean ± SEM. *p < 0.05, **p < 0.01, compared with Ctrl group, ^#^ *p* < 0.05, ^##^ *p* < 0.01 compared with the EtOH group. Significant differences between mean values were determined by one‐way ANOVA with Tukey's multiple comparisons test.

To investigate the ameliorating efficiency of UA on AICSD, we established a chronic alcohol consumption mouse model with an alcohol liquid diet and administered UA intervention for 16 weeks as described in animal experiment 1 (Figure 1A). There was no significant difference in body weight or food intake between the Ctrl group and the EtOH group as a result of pair‐feeding (Figure 1B,C). The Y‐maze test and the novel object recognition test were conducted to evaluate working memory and short‐term memory, respectively. Mice in the EtOH‐UA group exhibited an increased frequency of spontaneous alternations in the Y Maze, indicating that UA significantly ameliorated working memory deficits (60.43%) induced by alcohol consumption (*p* < 0.05; Figure 1D). Mice in the EtOH‐UA group also exhibited a higher discrimination index between the novel and familiar object without altered exploration time, implying that UA mitigated the short‐term memory deficits (12‐fold) induced by alcohol consumption (*p* < 0.01; Figure 1E,F). Additionally, the EtOH‐UA mice exhibited a lower escape latency (*p* < 0.01; Figure 1G) and a higher exploring frequency around the target hole (Figure S1G) compared to EtOH mice, indicating an improvement in long‐term memory (50.32%). Moreover, UA treatment improved sociability (10‐fold) and the preference for social novelty (12‐fold) compared to EtOH mice during the three‐chamber sociability test (*p* < 0.01; Figure 1H–K).

To distinguish between the preventive and therapeutic effects of UA, we further investigated its efficacy when initiated after long‐term alcohol exposure had been established. Following 8 weeks of chronic alcohol consumption, subsequent UA intervention significantly improved short‐term memory in the mice (*p* < 0.05). However, under this delayed intervention paradigm, UA administration did not lead to significant improvements in working memory, long‐term memory, or social function compared to the EtOH group.

In summary, concurrent UA administration from the onset of alcohol exposure effectively prevented the development of cognitive and social deficits. In contrast, initiating UA intervention after 8 weeks of established alcohol exposure provided a more limited benefit, rescuing only short‐term memory but no other behavioral domains.

### 2.2 UA Attenuates Alcohol‐Induced Synaptic Impairment and Neuroinflammation

We next sought to determine whether these behavioral impairments were associated with synaptic damage and neuroinflammatory responses in the brain. We found that UA intervention significantly ameliorated the alcohol‐induced morphological abnormalities in hippocampal neurons (Figure 1O). Next, we aimed to investigate the effect of UA on synaptic plasticity and neuroinflammation in AICSD mice. The observation of synaptic ultrastructure and analysis of postsynaptic density (PSD) revealed a significant increase in both the length and width of PSD in EtOH‐UA mice compared to the EtOH mice (*p* < 0.01; Figure 1L–N). Additionally, alcohol suppressed the expression of synaptic plasticity‐related genes (*Psd95* and *Fxr1*), neuronal development‐related genes (*Fxr2* and *Tdp2*), NMDA receptor gene *Glun2b*, and AMPA receptor gene *Glua2* in the hippocampus of murine brain in EtOH mice, which were significantly restored by UA treatment (*p* < 0.01; Figure S1F). Moreover, the protein expression of PSD‐95 (*p* < 0.01; Figure 1P,Q) was increased in the EtOH‐UA mice, whereas the expression of amyloid precursor protein (APP) was decreased compared to that in the EtOH mice (Figure S1H).

Considering the reported dysregulation of microglia in various neurodegenerative disorders [25], we assessed the expression of microglia activation‐related genes (*Iba1* and *Fyb*), microglia‐neuron interaction‐related genes (*Bdnf* and *Ngf*), and neuroinflammatory cytokine genes (*Il1b* and *Tnfa*) in the hippocampus. Chronic alcohol consumption increased the mRNA levels of these genes in the hippocampus of the EtOH mice, which were significantly restored by the UA treatment (*p* < 0.05; Figure S1E). Consistently, the neuroinflammation‐related proteins IBA‐1 (*p* < 0.05; Figure S1B,D,H), TNF‐α, and IL‐10 (Figure S1H) were significantly decreased in the EtOH‐UA group compared to the EtOH group. Additionally, EtOH‐UA mice showed an increase in the brain‐derived neurotrophic factor (BDNF) compared to the EtOH mice (*p* < 0.05; Figure S1A,C; Figure S1H).

Collectively, our results demonstrate that UA exerted a protective effect against chronic alcohol‐induced synaptic dysfunction and microglia overactivation in mice.

### 2.3 DRD2 Mediates the Beneficial Effect of UA on Alcohol‐Induced Cognitive and Social Behavioral Deficits

**FIGURE 2:**
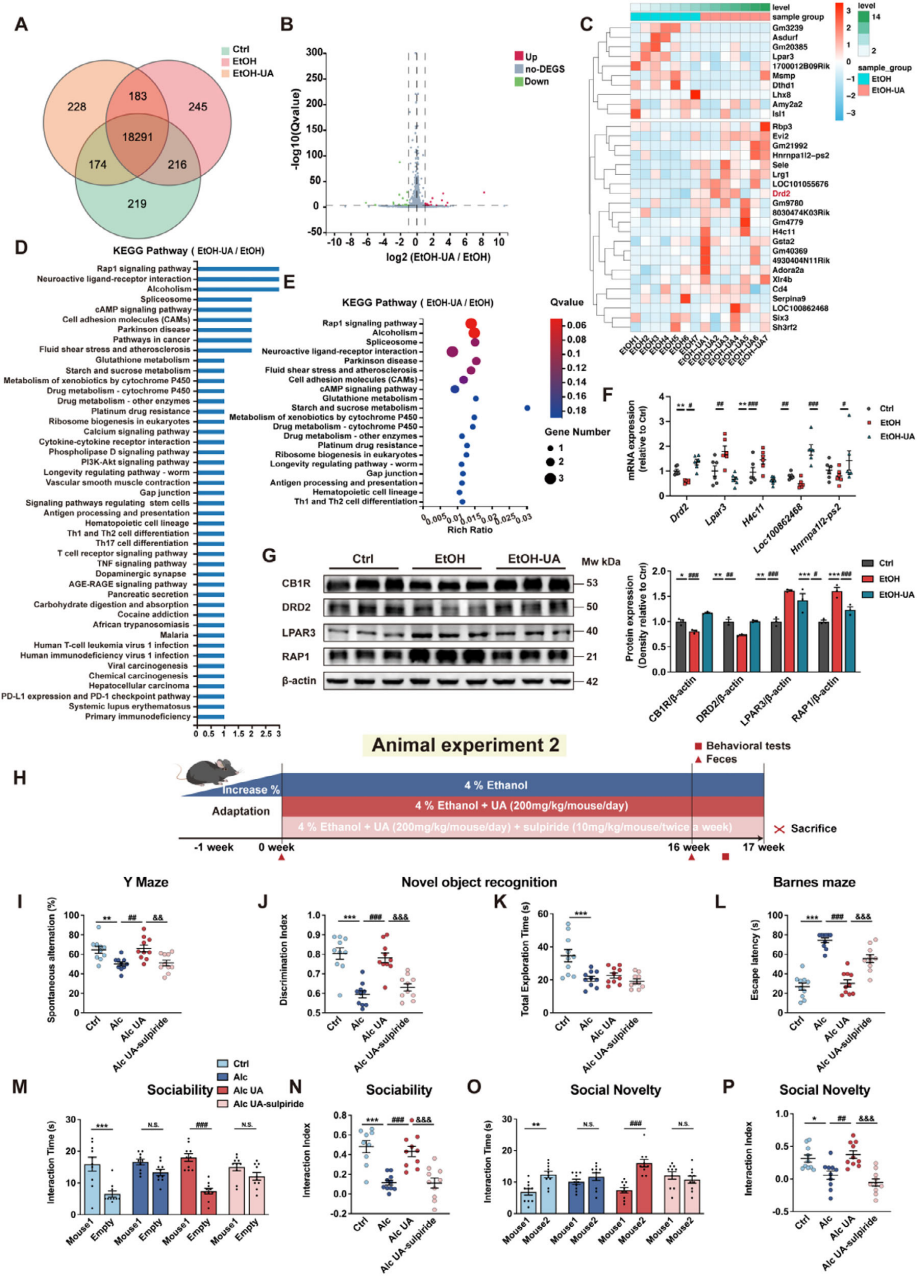
DRD2 mediates the effect of UA on alcohol‐induced cognitive and social behavioral deficits. (A) VENN plot, showing the differential expression of genes between different comparison groups. (B) Volcano plot based on the differential genes (EtOH‐UA vs. EtOH). (C) Heatmap of differential genes between EtOH and EtOH‐UA group based on the RNA sequencing of the hippocampus (*n* = 7, representative of 7 biological replicates for each group). (D) KEGG Pathway annotation and classification results of differential genes. (E) Bubble map of differential gene enrichment in KEGG Pathway. (F) The relative mRNA levels of differential genes between EtOH and EtOH‐UA groups based on the RNA sequencing of the hippocampus (*n* = 6 biologically independent samples per group). (G) Western blot analysis of differential genes between EtOH and EtOH‐UA group based on the RNA sequencing of the hippocampus (*n* = 3 biologically independent samples per group). (H) Timeline of animal experiment 2 depicting the alcohol liquid diet of UA and sulpiride treatment. (I) For the Y‐maze, spontaneous alternations were recorded (*n* = 10 mice per group). (J,K) For the novel object recognition test, the discrimination index between the novel and familiar objects and total exploration time was calculated (*n* = 10 mice per group). (L) For the Barnes maze, escape latency was recorded (*n* = 10 mice per group). (M) In the sociability test, the time spent interacting with a mouse or with an empty wire cage was recorded (*n* = 10 mice per group). (N) In the sociability test, the interaction index was calculated (*n* = 10 mice per group). (O) In the social novelty test, the time spent interacting with a novel versus a familiar mouse was recorded (*n* = 10 mice per group). (P) In the social novelty test, the interaction index was calculated (*n* = 10 mice per group). Data presented as mean ± SEM. *p < 0.05, **p < 0.01, compared with Ctrl group, ^#^ *p* < 0.05, ^##^ *p* < 0.01 compared with the EtOH group, ^&^ *p* < 0.05, ^&&^ *p* < 0.01 compared with the EtOH‐UA group. Significant differences between mean values were determined by one‐way ANOVA with Tukey's multiple comparisons test.

To investigate the global gene transcriptomics and identify the potential molecular targets underlying the beneficial effect of UA on AICSD, we performed RNA sequencing (RNA‐seq) analysis using hippocampal tissues from mice in the Ctrl, EtOH, and EtOH‐UA groups. The results indicate that 216 co‐regulated genes were identified between the EtOH group and the EtOH‐UA group (Figure 2A). Volcano plot analysis (Figure 2B) and hierarchical clustering heatmaps (FDR *q* < 5%, Figure 2C) were utilized to visualize differentially expressed genes (DEGs) in the hippocampus of mice from the EtOH group and the EtOH‐UA group. Notably, KEGG pathway analysis revealed that the *Drd2* gene was enriched in the Rap1 signaling pathway and the neuroactive ligand‐receptor interaction pathway (Figure 2D,E). The key differential genes were validated using quantitative real‐time PCR (qRT‐PCR), and the obtained results were consistent with the findings from RNA‐Seq analysis (*p* < 0.05; Figure 2F). Moreover, our findings indicate that UA enhanced the protein expression of DRD2 while concurrently reducing the protein expression of RAP1 (*p* < 0.05; Figure 2G), which indicates that DRD2 has the potential to regulate Rap1 signaling in line with the results of the hippocampal transcriptome. Dopamine signaling plays important roles in neural development and crucially contributes to brain functions encompassing locomotion, emotion regulation, learning processes, and memory formation [26, 27, 28]. Therefore, we hypothesized that UA protected against AICSD through the modulation of the DRD2 target.

To confirm the potential role of DRD2 in the beneficial effect of UA on AICSD, we designed the DRD2‐inhibited mouse model in animal experiment 2 (Figure 2H). Our findings demonstrate that inhibiting DRD2 significantly attenuated or even abolished the beneficial effects of UA on cognitive functions, including working memory, short‐term and long‐term memory (*p* < 0.01; Figure 2I–L), as well as social interaction in mice with chronic alcohol consumption (*p* < 0.01; Figure 2M–P). These findings demonstrate that DRD2 played a pivotal role in the amelioration mechanism of UA against AICSD.

### 2.4 UA Restores Alcohol‐Induced Dysbiosis of Intestinal Microecology

Alcohol consumption induces impairment of the intestinal barrier, leading to translocation of bacteria and bacterial endotoxins into the bloodstream and brain, thereby eliciting cognitive dysfunction and neuroinflammation [29]. To explore whether UA exerts its beneficial effect on AICSD by modulating the gut–brain axis, we investigated the impact of UA on intestinal barrier integrity in mice with chronic alcohol consumption. H&E staining and Alcian blue staining of colon tissues revealed that UA significantly alleviated alcohol‐induced epithelial damage and markedly increased the length of the colonic villi in EtOH‐UA mice compared to the EtOH mice (*p* < 0.01; Figure S4A,B). UA also increased the overall length of colon tissues (Figure S4D). Additionally, UA significantly enhanced the mRNA expression levels of intestinal tight junction proteins, intestinal mucins, and intestinal antimicrobial peptides in the colon of mice (Figure S4E–G). Moreover, UA significantly enhanced the expression of key tight junction proteins, including Occludin (Figure S4A,C), ZO‐1, and Claudin‐1 (Figure S4H,I). Furthermore, our findings demonstrate that UA significantly downregulated the mRNA expression levels of intestinal inflammatory factors and markedly reduced LPS levels in the serum of mice from the EtOH‐UA group (*p* < 0.01; Figure S4J,K), suggesting that modulating microbiota may play a critical role in the beneficial effects of UA on AICSD.

**FIGURE 3:**
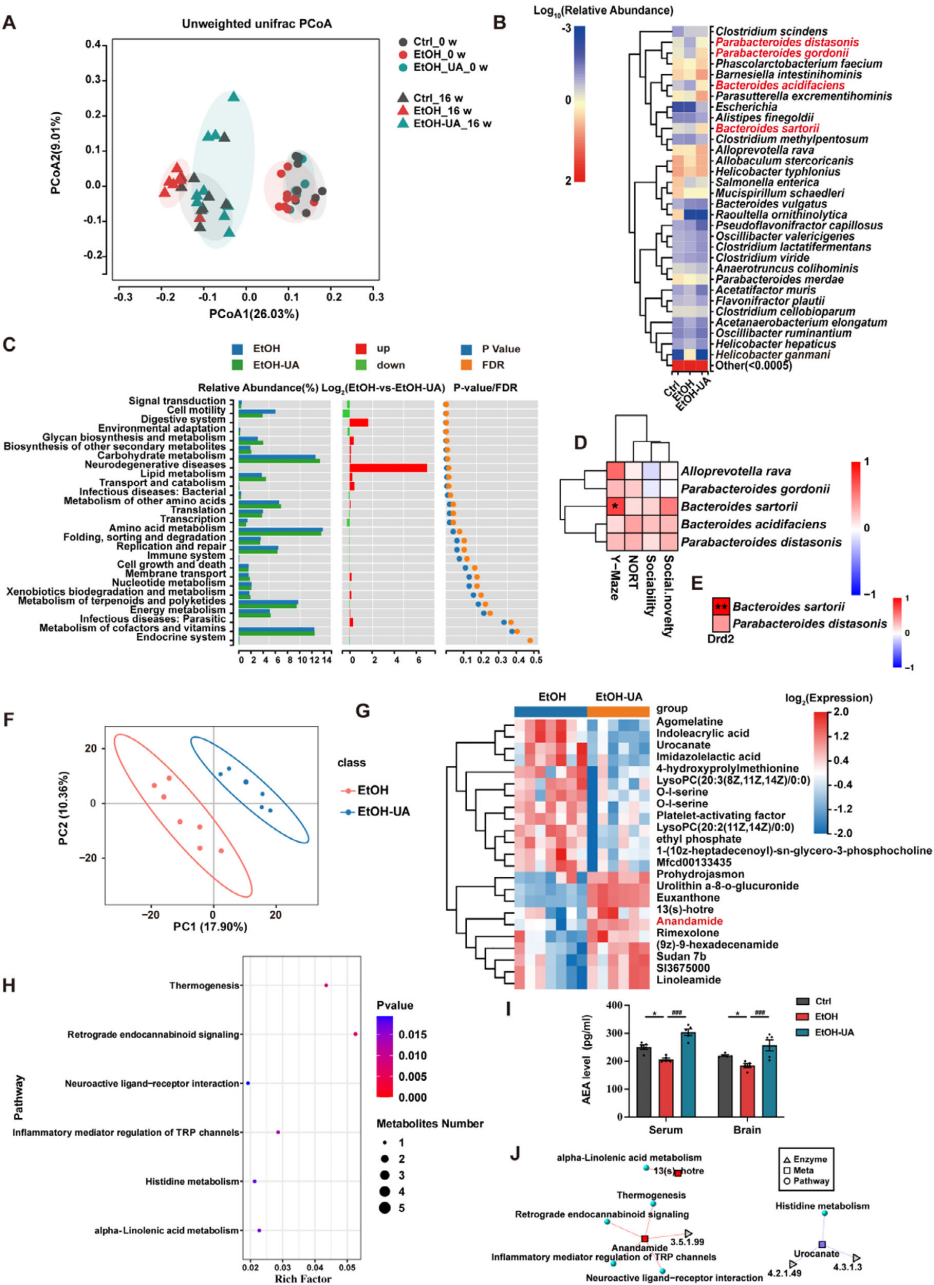
Gut microbiome and plasma metabolome analysis of UA‐treated alcoholic mice. (A) Principal coordinate analysis (PCoA) based on unweighted Unifrac distance and permutational manova (Adonis) were used to test the difference in gut microbiota composition and diversity between groups. (PERMANOVA, **p < 0.01) (B) Heatmap of the abundance of gut microbiota at the species level. (C) KEGG function enrichment of differential bacteria between the EtOH and EtOH‐UA groups. (D) Heatmap of correlation analysis between the cognitive and social impairment data and the relative abundance of UA‐enriched species. (The color intensity in the heatmap represents the Spearman's correlation coefficient, with asterisks indicating significance levels adjusted by the Holm correction: *p < 0.05, **p < 0.01, ***p < 0.001.)) (E) Heatmap of correlation analysis between Drd2 and the relative abundance of UA‐enriched species (*B. sartorii* and *P. distasonis)*. (The color intensity in the heatmap represents the Spearman's correlation coefficient, with asterisks indicating significance levels adjusted by the Holm correction: *p < 0.05, **p < 0.01, ***p < 0.001.) (F) PCA analysis of the distribution and separation trend of samples from EtOH‐UA and EtOH groups. (G) Heatmap of differential metabolites between EtOH and EtOH‐UA group (*n* = 6, representative of 6 biological replicates for each group). (H) Metabolic pathway enrichment analysis bubble chart. (I) The concentration of AEA in serum and brain. (J) Correlation heatmap of differential metabolites. Data presented as mean ± SEM. *p < 0.05, **p < 0.01, compared with Ctrl group, ^#^ *p* < 0.05, ^##^ *p* < 0.01 compared with the EtOH group. Significant differences between mean values were determined by one‐way ANOVA with Tukey's multiple comparisons test.

Increasing evidence has shown that dysbiosis of the intestinal microbiota plays a crucial role in the progression of alcohol‐related diseases [30]. To further decipher the underlying mechanism that UA protected against AICSD, we thus performed 16S rRNA sequencing to investigate the effects of UA on gut microbiota. As depicted in the principal coordinate analysis (PCoA) plot, the composition of the intestinal microbiome exhibited clustering among all three groups before the intervention (Figure 3A). However, at week 16, the composition of the intestinal microbiome in the EtOH‐UA mice clustered separately from that of EtOH mice, and displayed closer proximity to the Ctrl mice (Figure 3A). UA significantly improved the diversity (*p* < 0.01; Figure S3A) and richness (Figure S3B) of microbiota as well as β diversity (Figure S3C) in the EtOH‐UA mice compared to the EtOH mice. In addition, the EtOH‐UA group exhibited a higher abundance of *Bacteroidetes* compared to the EtOH group (Figure S3E–G). Notably, a comprehensive analysis revealed an elevation in the abundance of *B. sartorii* and *P. distasonis* in the EtOH‐UA group compared to the EtOH group (Figure 3B; Figure S3H). To elucidate the molecular mechanisms underlying the impact of microbial metabolism on cognitive function, we performed a functional difference analysis of microbial metabolic pathways and revealed a significant downregulation of the KEGG pathway associated with neurodegenerative diseases in EtOH‐UA mice compared to the EtOH mice (Figure 3C). To further investigate the relationship between alterations in microbiota and cognition or social impairments, we performed a correlation analysis to identify the association between microbial abundance and cognitive/social functions. The findings revealed a positive correlation between the abundance of UA‐enriched species and working memory, short‐term memory, and social behaviors (Figure 3D). We further analyzed the correlations between the UA‐regulated key gene *Drd2* and UA‐upregulated *B. sartorii* as well as *P. distasonis*. It was found that *Drd2* exhibited significant positive correlations with *B. sartorii* while also showing positive correlations with *P. distasonis* (Figure 3E).

To further elucidate the effects of UA microbiota metabolites, we performed comprehensive non‐targeted serum metabolomics profiling. Analytical results demonstrate that UA profoundly altered the composition and abundance of lipid‐related serum metabolites in the circulatory system (Figure 3F,G; Figure S5B). Metabolite pathway enrichment analysis revealed that the retrograde endocannabinoid signaling pathway, which was significantly enriched by the metabolite AEA, exhibited the most pronounced changes (Figure 3H). We quantified the levels of AEA and observed a significant elevation in AEA levels in the serum, brain (Figure 3I), and feces (Figure S5F) of mice following UA intervention.  Specifically, UA intervention in alcohol‐fed mice increased the levels of the AEA precursor linoleic acid while decreasing serum levels of arachidonic acid, which is derived from AEA hydrolysis (Figure S5E). Collectively, these results suggest that UA‐enriched gut microbiota and its associated metabolites may play vital roles in alleviating AICSD.

### 2.5 Gut Microbiota and Metabolites Mediate the Beneficial Effects of UA on Alcohol‐Induced Cognitive and Social Dysfunction

**FIGURE 4:**
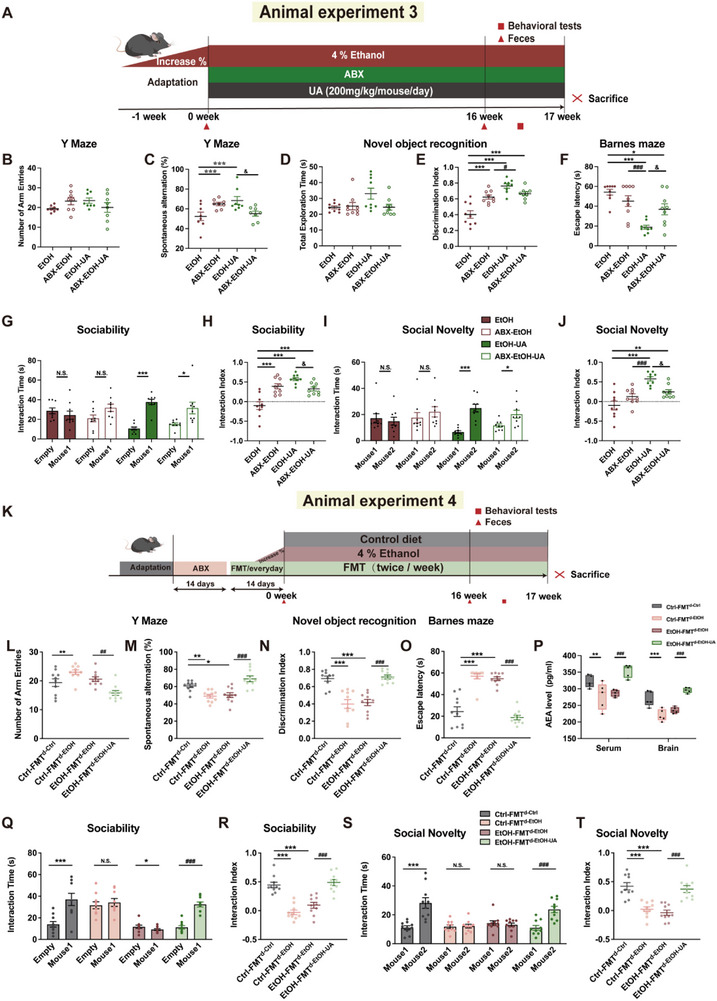
UA ameliorates alcohol‐induced cognitive and social behavioral deficits in a gut microbiota‐dependent manner. (A) Timeline of animal experiment 3 depicting the alcohol liquid diet of UA and the antibiotics treatment. (B,C) For the Y‐maze, spontaneous alternations and the number of arm entries were recorded. (*n* = 8 mice per group) (D,E) For the novel object recognition test, the exploration time and discrimination index between the novel and familiar objects were calculated (*n* = 9 mice per group). (F) For the Barnes maze, escape latency was recorded (*n* = 9 mice per group). (G) In the sociability test, the time spent interacting with a mouse or with an empty wire cage was recorded (*n* = 9 mice per group). (H) In the sociability test, the interaction index was calculated (*n* = 9 mice per group). (I) In the social novelty test, the time spent interacting with a novel versus a familiar mouse was recorded (*n* = 9 mice per group). (J) In the social novelty test, the interaction index was calculated (*n* = 9 mice per group). (K) Timeline of animal experiment 4 depicting the alcohol liquid diet with FMT treatment. (L,M) For the Y‐maze, spontaneous alternations and the number of Arm Entries were recorded (*n* = 10 mice per group). (N) For the novel object recognition test, the exploration time and discrimination index between the novel and familiar objects were calculated (*n* = 10 mice per group). (O) For the Barnes maze, escape latency was recorded (*n* = 10 mice per group). (P) The concentration of AEA in the serum and brain of FMT‐treated mice (*n* = 5 mice per group). (Q) In the sociability test, the time spent interacting with a mouse or with an empty wire cage was recorded (*n* = 10 mice per group). (R) In the sociability test, the interaction index was calculated (*n* = 10 mice per group). (S) In the social novelty test, the time spent interacting with a novel versus a familiar mouse was recorded (*n* = 10 mice per group). (T) In the social novelty test, the interaction index was calculated (*n* = 10 mice per group). For the antibiotic experiment, data were presented as mean ± SEM. *p < 0.05, **p < 0.01, compared with EtOH group, ^#^ *p* < 0.05, ^##^ *p* < 0.01, compared with ABX‐EtOH group, ^&^ *p* < 0.05, ^&&^ *p* < 0.01 versus EtOH‐UA group. Significant differences between mean values were determined by two‐way ANOVA (UA and antibiotics treatment as two factors) with Tukey's multiple comparisons test. For the FMT experiment, data were presented as mean ± SEM. *p < 0.05, **p < 0.01, compared with Ctrl‐FMT^d‐Ctrl^ group, ^#^ *p* < 0.05, ^##^ *p* < 0.01 compared with the EtOH‐FMT**^d‐ETOH^** group. Significant differences between mean values were determined by one‐way ANOVA with Tukey's multiple comparisons test.

To elucidate the pivotal role of gut microbiota in mediating the effects of UA on AICSD, we sought to investigate whether the beneficial effects of UA could be affected by the depletion of gut microbiota. An antibiotic cocktail treatment was administered in animal experiment 3 (Figure 4A). Our findings reveal that antibiotic treatment significantly blunted the beneficial effects of UA on working memory (Figure 4B,C), short‐term (Figure 4D,E), and long‐term memory (*p* < 0.05; Figure 4F), as well as the social ability of mice with chronic alcohol consumption (*p* < 0.05; Figure 4G–J). Moreover, our results showed that antibiotic treatment attenuated the improvement effect of UA on the excessive activation of microglia, neuroinflammation, and synaptic plastic impairment in chronic alcohol mice (Figure S6C). It is noteworthy that antibiotic treatment blunted the UA‐induced upregulation of *Drd2* expression, thereby reinforcing the findings that the effect of UA on DRD2 upregulation is dependent on the gut microbiota (Figure S6E). In parallel, we observed in non‐alcohol‐fed mice (animal experiment 9) that UA administration significantly enhanced working memory (*p* < 0.01; Figure S9C), while exerting minimal and statistically insignificant effects on short‐term memory, long‐term memory, and sociability (Figure S9D–H). Conversely, antibiotic treatment alone impaired learning, memory consolidation, and social interaction in these mice.

We performed an FMT experiment to further investigate whether the development of AICSD and the UA alleviation of AICSD are transmissible via gut microbiota. Ctrl‐FMT**^d‐EtOH^** mice exhibited impaired memory (Figure 4L–O) and social behaviors (Figure 4Q–T), whereas the EtOH‐FMT**^d‐EtOH‐UA^** mice showed improvement in these behavioral deficits. Interestingly, it was observed that the alterations in metaboliteAEA were effectively transmitted through FMT. Specifically, EtOH‐FMT**^d‐EtOH‐UA^** mice exhibited a significantly higher level of AEA in both serum and brain compared to the EtOH‐FMT**^d‐EtOH^** mice (*p *< 0.01; Figure 4P). Additionally, Ctrl‐FMT**^d‐EtOH^** mice exhibited morphological abnormalities in the cerebral neurons (Figure S6A), and microglia were overactivated compared to those in the Ctrl‐FMT**^d‐Ctrl^** mice (*p* < 0.05; Figure S6B,D). In contrast, these abnormalities were significantly alleviated in the EtOH‐FMT**^d‐EtOH‐UA^** mice compared to the EtOH‐FMT**^d‐EtOH^** mice. It was noteworthy that a similar upregulated level of DRD2 was observed in the EtOH‐FMT**^d‐EtOH‐UA^** mice, consistent with the donor EtOH‐UA mice, suggesting a potential role of gut microbiota and their‐derived metabolites in modulating DRD2 expression (*p* < 0.05; Figure S6F,G). Additionally, the Ctrl‐FMT**^d‐EtOH^** mice exhibited decreased protein expressions of PSD‐95 and BDNF, along with increased protein expressions of APP and BACE1 in the hippocampus of mice (Figure S6H). However, these protein expression levels were restored to normal in EtOH‐FMT**^d‐EtOH‐UA^** mice compared to the EtOH‐FMT**^d‐EtOH^** mice (Figure S6H).

In order to further verify the reliability of FMT, we further investigated the effect of FMT on gut barrier and gut microbiota in mice. We discovered that the Ctrl‐FMT**^d‐EtOH^** mice exhibited pronounced gut barrier damage compared to the Ctrl‐FMT**^d‐Ctrl^** mice, whereas the EtOH‐FMT**^d‐EtOH‐UA^** mice showed a significant improvement in gut barrier function compared to the EtOH‐FMT**^d‐EtOH^** mice (Figure S7A,B). After FMT intervention, a total of 347 common intestinal flora species were identified across the four groups of mice (Figure S7C). Notably, the PCoA analysis (Figure S7D) and the unweighted unifraccluster tree (Figure S7E) revealed that the gut microbiota of the Ctrl‐FMT**^d‐EtOH^** mice exhibited clustering patterns similar to those observed in the EtOH‐FMT**^d‐EtOH^** mice, whereas the gut microbiota of the EtOH‐FMT**^d‐EtOH‐UA^** mice displayed clustering patterns resembling those observed in the Ctrl‐FMT**^d‐Ctrl^** group, implying a similar microbiota diversity between these groups, respectively. Additionally, Ctrl‐FMT**^d‐EtOH^** mice also resulted in a reduction in the richness and diversity of gut microbiota. In contrast, EtOH‐FMT**^d‐EtOH‐UA^** mice exhibited an improvement in these parameters compared to the EtOH‐FMT**^d‐EtOH^** mice (Figure S7F). It is noteworthy that FMT exerts the most pronounced effect on the *Bacteroides* genus in mice (Figure S7G). Specifically, the abundance of *B. sartorii* and *P. distasonis* was significantly increased in EtOH‐FMT**^d‐EtOH‐UA^** mice compared to the EtOH‐FMT**^d‐EtOH^** mice (Figure S7H).

Collectively, these critical findings demonstrate that the alterations of cognitive and social functions were transmissible to the recipient mice along with FMT, highlighting the role of gut microbiota and their derived metabolites in mediating the neuroprotective effects of UA on AICSD.

### 2.6 Gut Bacteria Bacteroids Sartorii and Parabacteroids Distasonis Derived AEA to Ameliorate Alcohol‐Induced Cognitive and Social Behavioral Deficits

**FIGURE 5:**
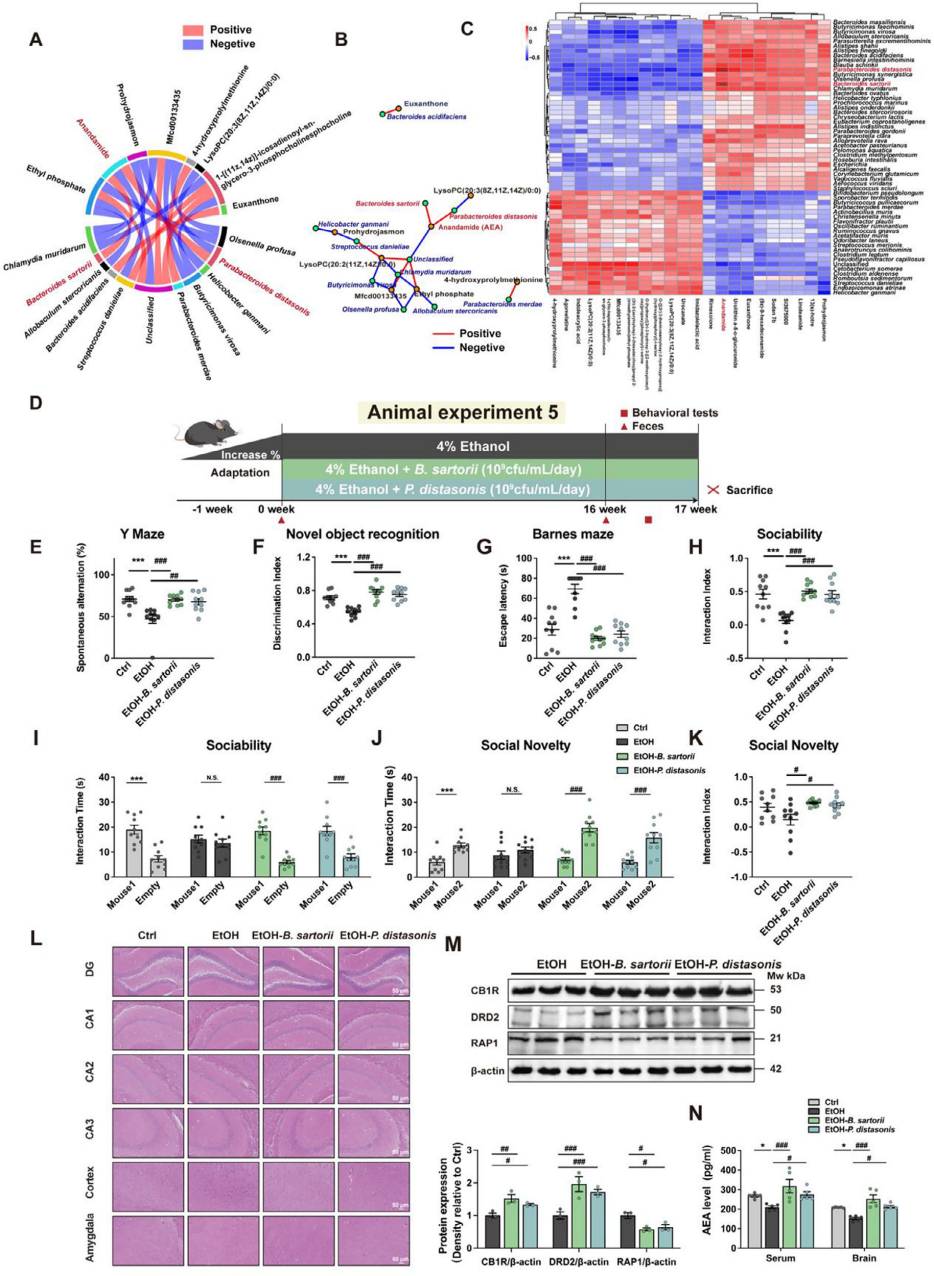
Gut bacteria *B. sartorii* and *P. distasonis* can enrich AEA to ameliorate alcohol‐induced cognitive and social dysfunction. (A) Spearman correlation chord diagram of microbial groups and differential metabolites. (B) Spearman correlation network interaction diagram of microbial groups and differential metabolites. (C) Spearman correlation heatmap of microbial groups and differential metabolites. (D) Timeline of animal experiment 5 depicting the alcohol liquid diet with *B. sartorii* and *P. distasonis* treatment. (E) For the Y‐maze, spontaneous alternations were recorded (*n* = 10 mice per group). (F) For the novel object recognition test, the discrimination index between the novel and familiar objects was calculated (*n* = 10 mice per group). (G) For the Barnes maze, escape latency was recorded (*n* = 10 mice per group). (H,I) In the sociability test, the time spent interacting with a mouse or with an empty wire cage was recorded (*n* = 10 mice per group). (J,K) In the social novelty test, the time spent interacting with a novel versus a familiar mouse was recorded (*n* = 10 mice per group). (L) Representative images of H&E staining of the brain and colon (*n* = 3 mice per group, scale bars, 50 µm). (M) Western blot analysis of DRD2, CB1R, and RAP1 in animal experiment 7. (N) The concentration of AEA in serum and brain (*n* = 5 mice per group). Data presented as mean ± SEM. *p < 0.05, **p < 0.01, compared with Ctrl group, ^#^ *p* < 0.05, ^##^ *p* < 0.01 compared with the EtOH group. Significant differences between mean values were determined by one‐way ANOVA with Tukey's multiple comparisons test.

To investigate the association between UA‐enriched microbiota and metabolites, we performed a correlation analysis between these two factors. The correlation between the overall microbiome and metabolites is illustrated using a correlation heatmap. Subsequently, subsets exhibiting high correlations are identified, and their regulatory expression patterns are elucidated through multidimensional analysis utilizing correlation network diagrams and chord diagrams. Our findings revealed a significant positive correlation between gut bacteria *B. sartorii, P. distasonis*, and the metabolite AEA (*p *< 0.01; Figure 5A–C). Thus, we hypothesized that UA‐enriched gut bacteria *B. sartorii* and *P. distasonis* could potentially enhance the level of AEA. To investigate this hypothesis, we conducted mono‐colonization experiments in animal experiment 5, where the alcohol‐fed mice were orally administered with cultured bacteria *B. sartorii* and *P. distasoni*s separately daily for 16 weeks (Figure 5D). Notably, we observed that both *B. sartorii* and *P. distasonis* significantly enhanced cognitive functions, including working memory (*p *< 0.01; Figure 5E), short‐term memory (*p *< 0.01; Figure 5F), and long‐term memory (*p *< 0.01; Figure 5G). Additionally, these strains improved sociability (*p *< 0.01; Figure 5H,I) and social novelty preference (*p *< 0.05; Figure 5J,K). Importantly, the therapeutic efficacy achieved by monocolonization with either strain was comparable to that of UA treatment itself (Figure S10), robustly supporting the conclusion that these bacteria are key mediators of UA's benefits.

Additionally, administration of *B. sartorii* and *P. distasonis* mitigated neuronal injury in the hippocampal CA1 and CA3 regions, as evidenced by reduced structural disorganization, improved cellular arrangement, decreased nuclear pyknosis, and attenuated morphological abnormalities (Figure 5L). Furthermore, our findings indicate that *B. sartorii* and *P. distasonis* have the potential to enhance the protein expression of CB1R (the receptor for AEA) and DRD2, while reducing RAP1 expression in the hippocampus of mice with alcohol consumption (Figure 5M). The noteworthy observation was that *B. sartorii* and *P. distasonis* significantly increased AEA levels in both serum and brain, indicating the potential of these bacteria to modulate AEA production in alcohol‐fed mice (Figure 5N). Taken together, the above results demonstrate that UA increased AEA derived from *B. sartorii* and *P. distasonis*, thereby contributing to its neuroprotective effects in mice with alcohol consumption.

### 2.7 AEA Mediates the Beneficial Effects of UA on Alcohol‐Induced Cognitive and Social Dysfunction via CB1R‐DRD2 Co‐Expression

**FIGURE 6:**
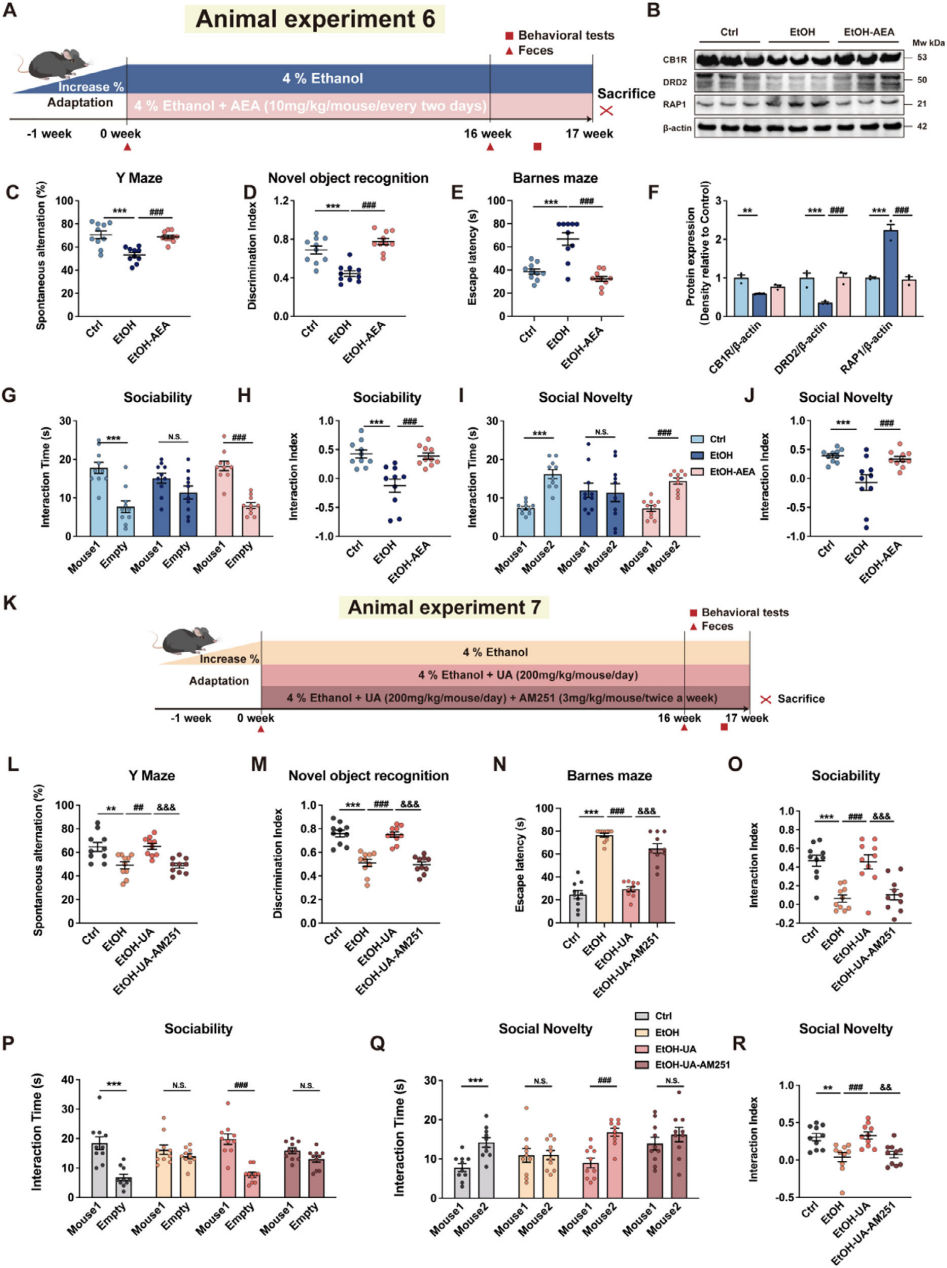
AEA‐CB1R mediates the effect of UA on alcohol‐induced cognitive and social behavioral deficits. (A) Timeline of experiment 6 depicting the alcohol liquid diet with AEA treatment. (B) Western blot analysis of DRD2, CB1R, and RAP1 in animal experiment 6. (C) For the Y‐maze, spontaneous alternations were recorded (*n* = 10 mice per group). (D) For the novel object recognition test, the discrimination index between the novel and familiar objects was calculated (*n* = 10 mice per group). (E) For the Barnes maze, escape latency was recorded (*n* = 10 mice per group). (F) Western blot analysis of DRD2, CB1R, and RAP1 in animal experiment 6 (*n* = 3) biologically independent samples per group). (G) In the sociability test, the time spent interacting with a mouse or with an empty wire cage was recorded (*n* = 10 mice per group). (H) In the sociability test, the interaction index was calculated (*n* = 10 mice per group). (I) In the social novelty test, the time spent interacting with a novel versus a familiar mouse was recorded (*n* = 10 mice per group). (J) In the social novelty test, the interaction index was calculated (*n* = 10 mice per group). (K) Timeline of animal experiment 7 depicting the alcohol liquid diet of UA and AM251 treatment. (L) For the Y‐maze, spontaneous alternations were recorded (*n* = 10 mice per group). (M) For the novel object recognition test, the discrimination index between the novel and familiar objects was calculated (*n* = 10 mice per group). (N) For the Barnes maze, escape latency was recorded (*n* = 10 mice per group). (O) In the sociability test, the interaction index was calculated (*n* = 10 mice per group). (P) In the sociability test, the time spent interacting with a mouse or with an empty wire cage was recorded (*n* = 10 mice per group). (Q) In the social novelty test, the time spent interacting with a novel versus a familiar mouse was recorded (*n* = 10 mice per group). (R) In the social novelty test, the interaction index was calculated (*n* = 10 mice per group). Data presented as mean ± SEM. *p < 0.05, **p < 0.01, compared with Ctrl group, **^#^** *p* < 0.05, **^##^** *p* < 0.01 compared with the EtOH group. Significant differences between mean values were determined by one‐way ANOVA with Tukey's multiple comparisons test.

Subsequently, we aimed to further investigate the role of AEA in mediating the effects of UA on AICSD by administering AEA intraperitoneally to alcohol‐fed mice in animal experiment 6 (Figure 6A). Consistent with the beneficial effects of UA treatment, the administration of AEA also significantly enhanced working memory (*p* < 0.01; Figure 6C), short‐term memory (*p* < 0.01; Figure 6D), and long‐term memory (*p* < 0.01; Figure 6E) in alcoholic mice. Furthermore, AEA treatment also improved sociability (*p* < 0.01; Figure 6G,H) and preference for social novelty (*p* < 0.01; Figure 6I,J). Moreover, our findings indicate that AEA enhanced the protein expression of CB1R and DRD2 while decreasing RAP1 in the hippocampus of alcohol‐fed mice (Figure 6B,F). To further clarify the underlying mechanism that AEA alleviates AICSD, we established the CB1R‐inhibited mouse model using the inhibitor AM251 in animal experiment 7. We treated the alcoholic mice with UA and administered an intraperitoneal injection of AM251 (3 mg/kg), a CB1R inhibitor for AEA, over a 16‐week period (Figure 6K). The results demonstrate that inhibition of CB1R significantly blunted the beneficial effects of UA on working memory (*p* < 0.01; Figure 6L), short‐term (*p* < 0.01; Figure 6M), long‐term memory (*p* < 0.01; Figure 6N), sociability (*p* < 0.01; Figure 6O,P) and social novelty preference in the EtOH‐UA‐AM251 mice (*p* < 0.01; Figure 6Q,R). Collectively, these data demonstrate that the beneficial effect of UA on AICSD depends on the activation of CB1R by AEA.

**FIGURE 7:**
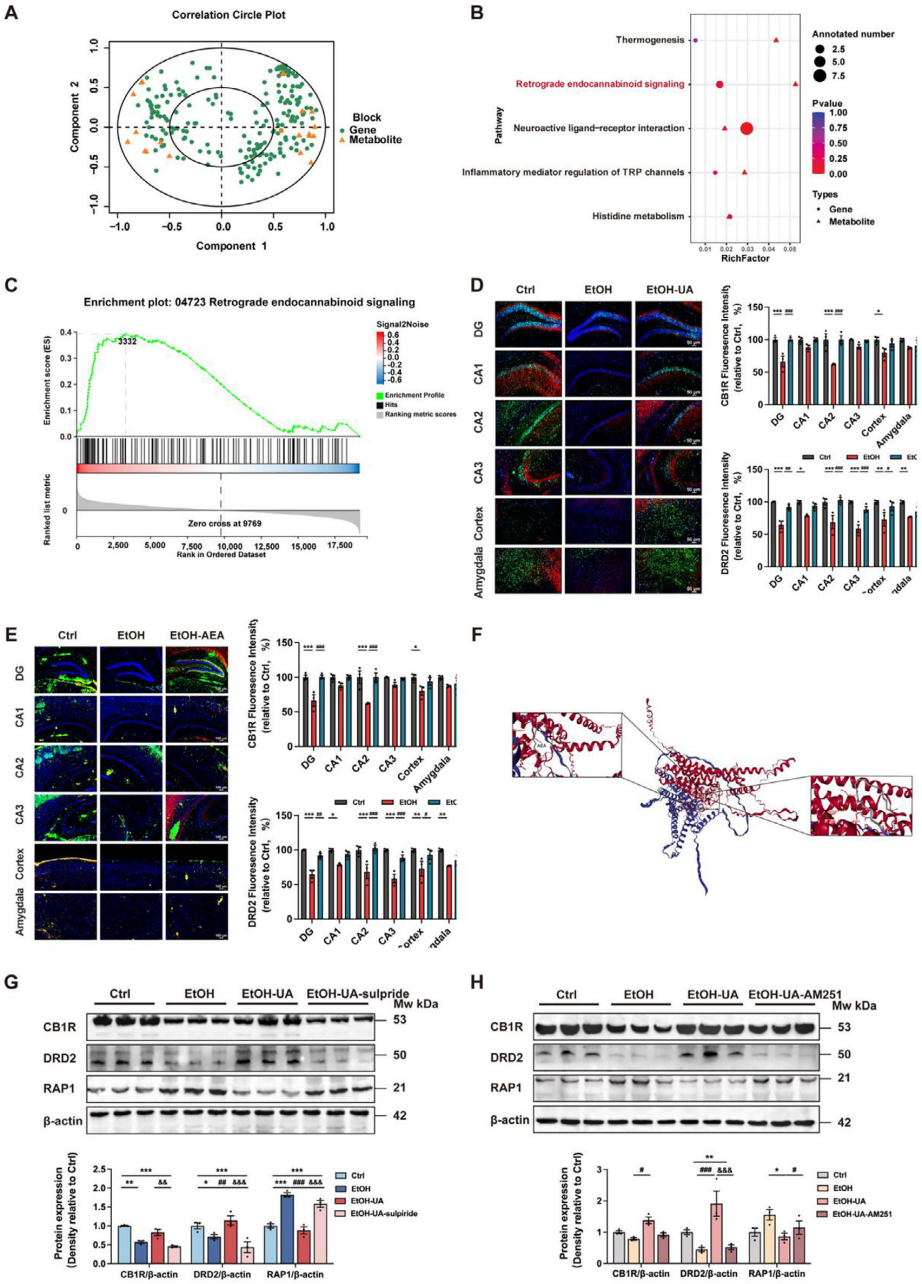
AEA mediates the beneficial effects of UA on alcohol‐induced cognitive and social dysfunction via CB1R‐DRD2 co‐expression. (A) Correlation Circle Plot for differential genes and differential metabolites in the EtOH‐UA group. (B) Bubble plot of differential gene and differential metabolic pathway enrichment analysis. (C) The KEGG pathway of retrograde endocannabinoid signaling was upregulated in the hippocampus of EtOH UA mice by GSEA. (D) Representative images and immunofluorescence intensity of CB1R and DRD2 in the hippocampus in animal experiment 1 (*n* = 3 mice per group, scale bars, 50 µm). (E) Representative images and immunofluorescence intensity of CB1R and DRD2 in the hippocampus in animal experiment 6 (*n* = 3 mice per group, scale bars, 100 µm). (F) Prediction diagram of molecular docking for AEA‐CB1R‐DRD2 simulation. (G) Western blot analysis of DRD2, CB1R, and RAP1 in animal experiment 2 (*n* = 3 biologically independent samples per group). (H) Western blot analysis of DRD2, CB1R, and RAP1 in animal experiment 6 (*n* = 3 biologically independent samples per group). Data presented as mean ± SEM. *p < 0.05, **p < 0.01, compared with Ctrl group, **^#^** *p* < 0.05, **^##^** *p* < 0.01 compared with the EtOH group, ^&^ *p* < 0.05, ^&&^ *p* < 0.01 compared with the EtOH‐UA group. Significant differences between mean values were determined by one‐way ANOVA with Tukey's multiple comparisons test.

Next, we performed a correlation analysis between metabolomics and transcriptomics data, revealing that differential genes and metabolites in the EtOH‐UA group were positively correlated (Figure 7A; Figure S8B,D). Moreover, metabolite AEA and transcription targets DRD2 were both enriched in the retrograde endocannabinoid signaling pathway (Figure 7B). Notably, the results of gene set enrichment analysis (GSEA) revealed that differential gene expression between the EtOH‐UA group and the EtOH group was significantly enriched in the retrograde endocannabinoid signaling pathway, which was upregulated upon UA treatment (Figure 7C). Thus, we speculated that AEA might be associated with DRD2 activation via its specific receptor CB1R in the hippocampus. Our findings demonstrate the co‐expression of CB1R and DRD2 in the hippocampus of EtOH‐UA mice (*p* < 0.05; Figure 7D). In accordance with the beneficial effects of UA treatment, the co‐expression of CB1R and DRD2 in the hippocampus of EtOH‐AEA mice was observed, indicating a potential collaborative regulatory role between these two receptors mediated by AEA (*p* < 0.05; Figure 7E). To further investigate the binding interactions between AEA, CB1R, and DRD2, molecular docking simulations were conducted as illustrated in Figure 7F. The results demonstrated that AEA binds to the binding pocket of the CB1R‐DRD2 complex, forming hydrophobic contacts with the ARG151 residue. Moreover, our results demonstrate that inhibiting the DRD2 blunted the beneficial effect of UA on enhancing CB1R (*p* < 0.01; Figure 7G). Similarly, inhibiting the CB1R also blunted the effect of UA on enhancing DRD2 (*p* < 0.01; Figure 7H). Notably, our findings demonstrate that inhibiting either DRD2 or CB1R attenuated the UA‐induced downregulation of RAP1 (Figure 7G,H), implying that the co‐expression of DRD2 and CB1R may exert inhibitory effects on RAP1 signaling.

Collectively, these results indicate that AEA mediated the beneficial effects of UA on alcohol‐induced cognitive and social behavioral deficits via the CB1R‐DRD2‐RAP1 signaling axis.

## 3 Discussion

Chronic excessive alcohol consumption ranks as the third major global health risk and is identified as the most significant factor contributing to early‐onset dementia, thus posing a substantial challenge to global public health [1, 2]. Therefore, it is imperative to develop more effective preventive and therapeutic interventions for addressing this global health challenge. UA, as a promising natural compound, has caught lots of attention due to its bioactivity in antiaging, anti‐AD, and neuron‐protection research [17, 31, 32]. However, the potential of UA in alleviating AICSD and the underlying mechanism involving gut microbiota remain elusive, highlighting the urgent necessity to be further investigated. Here, our research not only demonstrates the beneficial effects of UA on AICSD but also elucidates the underlying causal mechanism through the specific gut microbiota–endocannabinoids–brain axis. Multi‐omics combined analysis was utilized to identify the key gut microbiota, core metabolites, and response genes in the brain involved in improving AICSD by UA. Furthermore, research approaches including FMT, validation of core functional bacteria, metabolite supplementation, and specific blockade of target pathways, were utilized to elucidate the causal mechanisms by which UA enhances cognitive and social function via the gut microbiota–endocannabinoids–brain axis in mice with chronic excessive alcohol consumption. Finally, we demonstrate that UA enriched the abundance of beneficial gut microbiota species *B. sartorii*, *P. distasonis*, and their derived AEA, thereby targeting the CB1R‐DRD2‐RAP1 signaling axis to ameliorate AICSD.

Excessive alcohol consumption exerts detrimental effects on neurotransmitter systems, increases the permeability of the blood–brain barrier (BBB), thereby inducing neuronal cell apoptosis, impairing the glial function, and ultimately resulting in brain damage [33, 34]. Here, we demonstrate that UA can prominently ameliorate AICSD, alleviate neuroinflammation, and enhance synaptic plasticity in mice with alcohol consumption. Interestingly, the results of animal experiment 8 revealed that delayed UA intervention also exhibited a mitigating effect of AICSD; however, its efficacy was significantly inferior to that of early intervention, implying the protective effects of UA against AICSD and a superior efficacy associated with earlier initiation of UA intervention (Figure S2). Our study demonstrates that the ameliorative effect of UA on AICSD is mediated by the specific target DRD2 (Figure 2). DRD2, as a G protein‐coupled receptor, exerts regulatory effects on synaptic transmission and plays a pivotal role in various cognitive functions of the brain, and has long been recognized as a therapeutic target for mental disorders [35, 36]. Notably, the present study demonstrates that UA effectively alleviates AICSD mediated by DRD2 in a microbiota‐dependent manner. Gut microbiota plays a crucial role in maintaining the balance of the dopamine system involving the alterations in the expression of dopamine transporter (DAT) and DRD1 or DRD2, through intricate and bidirectional communication, known as the microbiota–gut–brain axis [37, 38]. In line with our findings, a preclinical study revealed a correlation between microbiome alterations and decreased expression of dopamine DRD2 mRNA, which is associated with increased severity of alcohol use disorder [38]. Collectively, these results highlighted the crucial role of microbiota in UA modulation of DRD2, thereby providing protection against AICSD.

Alcohol consumption can induce intestinal microbiota dysbiosis via multiple mechanisms, specifically an imbalance in the intestinal microbial community. Chronic alcohol exposure can decrease the production of antimicrobial peptides, including mucus and α‐defensins, and compromise the integrity of the intestinal barrier [39, 40]. Alcohol consumption can also modify the gut microbiota by decreasing the production of short‐chain fatty acids, which are beneficial metabolic products of microbial fermentation [41]. Research has demonstrated that alcohol intake can result in bacterial overgrowth and dysbiosis. Specifically, alcohol can decrease the abundance of *Bacteroides*, *Clostridia*, and Proteobacteria, while simultaneously increasing the prevalence of *Pseudomonas*, Gamma‐Proteobacteria, and Bacilli [42]. Alcohol abuse induces dysbiosis in the intestinal microbiota, leading to impaired CNS functionality that manifests as cognitive dysfunction and mood alterations [43]. Previous studies have provided compelling evidence for the efficacy of probiotic treatment in establishing a strong association between neuropsychiatric disorders induced by alcohol use disorder and the gut–microbiota–brain axis [43]. Herein, we observed that UA enhanced gut barrier integrity and microbial homeostasis with decreased plasma LPS level, providing critical insights into the mechanisms underlying the neuroinflammatory response modulation by UA. Our results demonstrate that UA‐enriched beneficial bacteria, such as *B. sartorii* and *P. distasonis*, exerted a positive correlation with the improvement of AICSD (Figure 3). There is a growing appreciation that the enrichment of *B. sartorii* has the potential to ameliorate non‐alcohol fatty liver disease and its related metabolic syndrome [44], retarding aging processes and preventing age‐related diseases [45]. Recent evidence supported the notion that humans with metabolic syndrome exhibit a diminished relative abundance of *P. distasonis* [46]. *P. distasonis* possesses the ability to modulate both neuroinflammation and brain barrier function, thereby mitigating cognitive impairment [47, 48]. Collectively, *B. sartorii* and *P. distasonis* demonstrated pivotal roles in the beneficial effects of UA on AICSD.

The restructuring of the gut microbiota by UA resulted in modifications to the microbial metabolites in plasma, potentially providing further insight into the mechanisms underlying the improvement of cognitive and social functions observed in mice with alcohol consumption. UA significantly altered a multitude of lipid‐related metabolites, with a predominant enrichment observed in lipid metabolism pathways (Figure S5). Our study revealed that UA significantly elevated serum levels of the endocannabinoid AEA in alcohol‐consuming mice. Crucially, our quantification reveals that this elevation occurs not only systemically in serum and brain but also locally within the intestinal lumen, as evidenced by the significant increase in fecal AEA (Figure S5F). This coordinated rise across compartments provides direct evidence that the gut is a primary site of AEA upregulation following UA intervention, effectively addressing the question of intestinal origin. Furthermore, UA not only increased the levels of the linoleic acid, a precursor of AEA, but also decreased the serum levels of arachidonic acid, which is derived from AEA hydrolysis, in mice with alcohol consumption (Figure S5E). Such alterations in fatty acid levels may potentially arise from altered gut permeability or gut microbiota‐induced changes in lipid metabolism. Chronic alcohol consumption is a persistent stressor to the body, leading to a significant reduction in AEA levels. A recent study substantiated the notion that chronic stress is characterized by a decrease in AEA levels [49]. Clinical evidence has demonstrated that elevated AEA levels facilitate the retrieval of fear‐extinction memories and alleviate the anxiogenic consequences induced by stress, ultimately affecting synaptic function and behavioral abnormalities [50, 51]. Collectively, UA‐enriched *B. sartorii* and *P. distasonis* and metabolite AEA may possess potential neuroprotective properties to ameliorate AICSD.

We further employed antibiotic treatment and FMT to validate the mediating function of the gut microbiota and metabolites in UA ameliorating AICSD. The beneficial effects of UA on AICSD were suppressed after removing the gut microbiota in mice with alcohol consumption through antibiotic treatment (Figure 4), implying a pivotal role of microbiota composition and their derived metabolites in mediating the neuroprotective effects of UA. It is noteworthy that antibiotic treatment diminished the upregulation of UA on DRD2 in the hippocampus, further supporting the robust correlation between gut microbiota and hippocampal DRD2 expression. Moreover, we observed a significant enhancement in the working memory of mice with a non‐alcoholic diet following the administration of UA in animal experiment 9, while its impacts on short‐term and long‐term memory as well as social ability were found to be minimal and statistically insignificant (Figure S9). Conversely, antibiotic treatment exhibited detrimental effects on learning, memory consolidation, and social interaction in mice fed with a non‐alcoholic diet (Figure S9). Additionally, our FMT experiment provided compelling evidence supporting the potential involvement of gut microbiota and metabolites in both the pathogenesis of AICSD and the alleviation effect of UA on AICSD, thereby highlighting the role of gut microbiota exert influence on the intricate functioning of remote organs. Our findings indicate that Ctrl‐FMT**^d‐EtOH^** mice exhibited significant cognitive and social behavioral deficits, which were associated with a reduced abundance of *B. sartorii* and *P. distasonis*, as well as decreased levels of AEA. These results suggest a potential involvement of gut microbiota and metabolites in the pathogenesis of AICSD. It is noteworthy that EtOH‐FMT**^d‐EtOH‐UA^** mice showed improved cognitive and social abilities. Additionally, the abundances of beneficial species *B. sartorii* and *P. distasonis*, as well as the metabolite AEA, were also significantly increased (Figure 4; Figure S7). The dysbiosis of gut microbiota in patients with alcohol use disorder and the presence of specific microbial communities have been implicated in both alcohol consumption and cognitive function [52]. To the best of our knowledge, this study has demonstrated for the first time that the beneficial effect of UA on AICSD can be transmitted via FMT, mediated by the key gut microbiota and AEA. Collectively, our findings not only elucidate novel microbiota‐dependent mechanistic pathways through which UA ameliorates AICSD, but also identify gut microbiota and associated metabolites as promising therapeutic targets for developing effective intervention strategies in preclinical models.

Our study establishes a significant positive correlation between the UA‐enriched gut bacteria B. sartorii and P. distasonis and the endocannabinoid AEA. Critically, functional monocolonization experiments confirmed that these strains are not merely associative but are functionally sufficient to elevate AEA levels and ameliorate AICSD. Notably, a comparative NCBI BLAST analysis revealed that the commercial strains used share a high degree of 16S rRNA identity with murine fecal isolates. This sequence conservation provides strong genomic support for their functional relevance. Moreover, as shown in Figure S10, the therapeutic effects achieved by monocolonization with either *B. sartorii* or *P. distasonis* were comparable to those of UA treatment itself, robustly supporting the conclusion that these specific bacterial strains are key mediators of UA's efficacy. These findings are supported by the established role of the endocannabinoid system in gut–brain communication [53]. Given the pivotal role of microbiota in the gastrointestinal tract, alterations in the gut microbiota can exert an influence on the homeostasis of endocannabinoid system balance and subsequently the integrity of the intestinal barrier [54]. Consequently, the endocannabinoid system is influenced by prebiotics, probiotics, and antibiotics [55]. Administration of bacterial strains may potentially enhance endocannabinoid synthesis by addressing microbial dysbiosis [53]. In our study, we initially discovered that oral administration of UA‐enriched gut bacteria *B. sartorii* and *P. distasonis* leads to an increase in AEA levels. The gut microbiota modulated by UA likely enhances AEA production through bacterial enzymatic activities and metabolic regulation of fatty acid pathways. For instance, recent studies revealed that *Gilliamella apicola*, a gut bacterium expressing Δ‐6 desaturase FADS2, facilitates the conversion of linoleic acid to arachidonic acid—a crucial precursor for AEA synthesis [56]. Similarly, *P. distasonis* has been shown to enhance γ‐linolenic acid metabolism, potentially contributing to fatty acid‐derived endocannabinoid biosynthesis [57]. Furthermore, emerging evidence suggests that specific gut microbes, such as *Lactobacillus*, can activate the endocannabinoid system via gut–brain axis signaling, as demonstrated by its ability to alleviate depressive symptoms in mice through hippocampal neurogenesis and upregulated endocannabinoid activity [58]. These findings collectively imply that UA‐enriched microbiota may promote AEA synthesis through direct enzymatic contributions, modulation of host fatty acid metabolism, and cross‐talk with enteroendocrine or neuroendocrine pathways.

However, the precise molecular mechanisms underlying these interactions remain incompletely elucidated, presenting a key direction for future research. Two central questions are paramount: first, the molecular mechanisms by which UA specifically promotes the growth of *B. sartorii* and *P. distasonis*; and second, how these two bacteria directly elevate host AEA levels. To address these questions, future studies should aim to determine whether UA serves as a direct nutrient source or a signaling molecule for these bacteria using transcriptomics and metabolomics. Concurrently, it will be crucial to identify whether bacterial‐derived factors directly interact with host enzymes involved in AEA biosynthesis (e.g., N‐acylphosphatidylethanolamine‐specific phospholipase D) or degradation (e.g., fatty acid amide hydrolase). Addressing these questions through combined bacterial genetic manipulation and host–cell co‐culture models will be essential to fully unravel the mechanistic dialogue within this gut–brain axis. Ultimately, elucidating these pathways will solidify the rationale for targeting the endocannabinoid system with precise microbial interventions as a promising strategy to improve AICSD in individuals with chronic alcoholism.

In our quest for the underlying mechanisms behind these dysfunctions, we unraveled that UA modulated fatty acid metabolism by enriching the neurotransmitter AEA to activate CB1R, thereby eliciting stimulation of the endocannabinoid system. We directly supplemented AEA to mice and discovered that AEA could prominently ameliorate AICSD. It has been reported that AEA exhibits anti‐inflammatory effects through the activation of cannabinoid receptors CB1R located on neurons, microglia, and astrocytes [59]. AEA also restores seizure‐induced impairment of short and long‐term synaptic plasticity in rats [60]. Our findings revealed that AEA administration effectively enhanced the expression of CB1R, thereby improving cognitive and social functions in alcohol‐fed mice (Figure 7). The modulation of endocannabinoid signaling through AEA has been proposed as a promising therapeutic target for various disorders related to emotions [61]. The endocannabinoid system is a neuro‐modulatory system within the brain that intricately regulates neuronal excitability and synaptic plasticity [62, 63]. Our results demonstrate that inhibiting the CB1R blunted the beneficial effects of UA on AICSD, thereby implying the crucial role of the endocannabinoid system in the neuroprotective properties of UA against AICSD. Considering the previous evidence illustrated anxiolytic and antidepressant‐like effects resulting from CB1R receptor activation, particularly through hippocampal neurogenesis modulation, we investigated more details of the brain endocannabinoid system. Seemingly distant, yet deeply intertwined, the social brain, the endocannabinoid system, and the gut microbiome constitute a complex matrix that deserves further investigation in AICSD.

Our results further indicate that AEA mediates the amelioration effects of UA on AICSD through the activation of the CB1R‐DRD2 co‐expression network (Figure 7). Association analysis of the metabolome and transcriptome revealed that AEA exhibited co‐enrichment with DRD2 in the retrograde endocannabinoid signaling pathway. CB1R, which serves as the receptor of AEA, was found to co‐express with DRD2 in the hippocampal region of the brain in EtOH‐UA and EtOH‐AEA mice (Figure 7). CB1R and DRD2 are G protein‐coupled receptors that couple to inhibitory Gαi/o proteins, leading to the suppression of cAMP production upon receptor activation [64]. The cannabinoid‐dopamine cross‐talk carries significant neurobiological implications, particularly given their convergent roles in modulating cognitive functions (learning and memory) and reward circuitry [65]. Several studies have demonstrated that cannabinoids can induce dopamine release in the brain, and it is well‐established that DRD2 is involved in the potentiation of arachidonic acid release [66, 67]. Additionally, CB1R knockout models display significantly enhanced binding affinity for the DRD2 antagonist raclopride [68, 69], with emerging evidence suggesting endocannabinoid‐mediated regulation of DRD2 activation thresholds [70]. In addition to the formation of heterodimers, there exists compelling evidence supporting functional interplay between CB1R and DRD2. Notably, emerging evidence indicates that their functional interplay may involve a direct protein–protein interaction [71]. Combined in situ hybridization, immunocytochemistry, and electron microscopy studies have revealed CB1R‐DRD2 co‐localization in the caudate putamen and nucleus accumbens [68, 69]. Our findings highlight that the ameliorative effects of UA on AICSD involve a synergistic interaction between the endocannabinoid and dopamine systems. Specifically, UA elevates endogenous AEA, a core metabolite of the endocannabinoid system, which binds to its cognate receptor CB1R. This activation of CB1R initiates cross‐talk with the dopamine system by co‐regulating DRD2 expression. Importantly, our data suggest that CB1R‐DRD2 co‐expression, rather than isolated activation of either receptor, is essential for mediating UA's therapeutic effects. CB1R serves as a molecular bridge that links AEA signaling to dopaminergic pathways, while DRD2 acts as the effector molecule responsible for restoring cognitive and social function. Experimental evidence, including receptor co‐expression analyses and functional blockade studies (animal experiment 2 and animal experiment 7), supports the necessity of this cooperative mechanism.

Our findings reveal a direct molecular link between AEA and RAP1 signaling that is contingent upon the CB1R‐DRD2 interaction. Critically, disruption of the CB1R‐DRD2 interaction completely abolishes AEA‐induced RAP1 activation.  This functional evidence, together with our structural data showing AEA bound to the CB1R‐DRD2 complex (e.g., through hydrophobic contacts with ARG151), collectively establishes the CB1R‐DRD2 complex as an essential signaling hub. This positions the CB1R‐DRD2 itself as the essential signaling platform. We propose a model in which AEA binding induces a conformational change in the CB1R‐DRD2 complex, allowing it to directly recruit and activate a specific RAP1 guanine nucleotide exchange factor (RAP1GEF, such as EPAC or C3G). Thereafter, the recruited RAP1GEF catalyzes the exchange of GDP for GTP on RAP1, leading to its activation. This mechanism defines the CB1R‐DRD2 complex as the direct molecular bridge from AEA engagement to RAP1 activation in AICSD. Given that stress‐mediated dysregulation of RAP1 impairs hippocampal structure and function [72], our proposed pathway, in which UA elevates AEA to activate CB1R‐DRD2 and subsequently RAP1, offers a precise molecular explanation for how the endocannabinoid‐dopamine cross‐talk enhances synaptic function and alleviates AICSD.

Moreover, the discovery of biomarkers holds great potential in the study of human neurodegenerative diseases, facilitating the development of rapid and non‐invasive diagnostic or prognostic approaches for clinical practice [73, 74]. Through the analysis of the supervised model combined with omics data, a model can be established to screen out the biomarker panel of microbial taxa, metabolites, and genes for differentiating biological phenotypes. By comparing the ROC curve (Receiver Operating Characteristic curve) among the separate modeling of different omics and the combined data modeling, which omics can better separate the control group and the experimental group was evaluated (Figure S8D,E). Our findings demonstrate that the gut microbiota, metabolite, and transcriptional target enriched with UA exhibited a remarkable ability to distinguish individuals with AICSD from the health controls, achieving an area under the receiver operating characteristic curve (AUROC) of 1.0 (Figure S8). These findings suggest that these biomarkers hold great potential as assessment methods for evaluating the efficacy of anti‐AICSD therapeutics.

The present study unveils a multitude of innovative findings. First, we demonstrate for the first time that UA ameliorated AICSD in a microbiota‐dependent manner, providing the irreplaceable role of microbiota in the beneficial effects of UA. Second, our findings indicate that UA can enrich the abundance of specific gut bacteria *B. sartorii* and *P. distasonis*, thereby elevating neurotransmitter AEA and providing innovative preventive and therapeutic approaches targeting the gut microbiota to alleviate alcohol‐induced neural damage. Further investigations are warranted to elucidate the underlying mechanism through which UA enhances the colonization of *B. sartorii* and *P. distasonis*, as well as the mechanism by which both bacteria contribute to an increase in AEA levels. Last, our results reveal that UA‐enriched AEA ameliorated AICSD through CB1R‐DRD2 signaling, thereby highlighting its potential as an effective intervention for AICSD.

Our study has some limitations that should be noted. While investigating the role of DRD2 receptors, this study employed the DRD2 receptor antagonist sulpiride. Future research should incorporate DRD2 knockout models to validate mechanisms with greater specificity. Additionally, this study exclusively used male mice, whereas alcohol metabolism and gut microbiota exhibit sex‐specific differences. Subsequent investigations will incorporate female models to enhance the comprehensiveness and validity of the research. Furthermore, it is important to acknowledge limitations in our microbial experimental design. The mechanistic validation relied on standard laboratory strains, which may not fully represent the functional dynamics of diverse endogenous microbial communities in natural or clinical settings. Finally, our in vivo intervention was conducted at a single dose of UA. Future studies employing dose‐response designs would be essential to define the therapeutic window and to strengthen the generalizability of our conclusions.

Overall, this study provides compelling evidence supporting the UA intervention as a novel and effective approach for managing alcohol‐related neurodegenerative diseases via the gut microbiota–brain axis, thereby addressing a critical challenge in the field of alcohol‐associated disorders. Notably, this finding opens up a novel and compelling association between gut microbiota and cognitive as well as social functions, elucidating the role of microbiota and metabolites in this association under UA treatment. Essentially, our research demonstrates for the first time that UA enhanced the abundance of beneficial species *B. sartorii* and *P. distasonis*, which enriched neurotransmitter AEA to activate endocannabinoid and dopamine systems, eventually ameliorating AICSD. Furthermore, the cognition‐related gut microbes, metabolites, neurotransmitters, and signaling pathways modulated by UA in this study may potentially contribute to the future development of clinical interventions or drug targets for AICSD and other neurodegenerative diseases.

## 4 Methods

### 4.1 Animal Experiments

Male C57BL/6J mice, aged 6 weeks at the time of acquisition, were obtained from the Cheng Du Dossy Laboratory Animal Center in China. The mice were housed in a specific pathogen‐free (SPF) laboratory environment, with four mice per cage. They were maintained in rooms with controlled humidity and temperature, following a 12‐hour light‐dark cycle. The study protocol adhered to the international ethical guidelines and was approved by the Institutional Animal Care and Use Committee of the Institute of Biomedical Research at Northwest A&F University of Technology, with ethics approval number XN2023‐0518.

### 4.2 Animal Experiment 1: UA Treatment Experiment

Male C57BL/6J mice (6 weeks old, SPF) were randomly assigned to three groups (*n* = 10 per group). The sample size per group was chosen based on common practices in comparable behavioral studies and considerations of the 3R principles to minimize animal use [75]. (1) Ctrl group: mice were fed an AIN93M control liquid diet and orally gavaged daily with sterilized CMC‐Na (0.05%); (2) EtOH group: mice were fed an AIN93M ethanol liquid diet and orally gavaged daily with sterilized CMC‐Na (0.05%); (3) EtOH‐UA group: mice were fed an ethanol liquid diet and orally gavaged daily with UA (200 mg/kg/day) dissolved in sterilized CMC‐Na (0.05%). The dose of UA was cited in accordance with our previous research [76, 77, 78]. The mice were euthanized following a 16‐week treatment period and a 1‐week behavioral test, after which brain and colon specimens were collected for qPCR analysis, western blot analysis, and histological evaluations.

### 4.3 Animal Experiment 2: DRD2 Inhibition Experiment

Male C57BL/6J mice (6 weeks old, SPF) were randomly assigned to four groups (*n* = 10 per group): (1) Ctrl group: mice were fed an AIN93M control liquid diet and orally gavaged daily with sterilized CMC‐Na (0.05%). Additionally, they received intraperitoneal injections of sterilized saline twice a week; (2) EtOH group: mice were fed an AIN93M ethanol liquid diet and orally gavaged daily with sterilized CMC‐Na (0.05%), and treated with intraperitoneal injections of sterilized saline twice a week; (3) EtOH‐UA group: mice were fed an AIN93M ethanol liquid diet and orally gavaged daily with UA at a dosage of 200 mg/kg/day, and treated with intraperitoneal injections of sterilized saline twice a week; (4) EtOH‐UA‐sulpiride group: mice were fed an AIN93M ethanol liquid diet and orally gavaged daily with UA (200 mg/kg/day), and treated with intraperitoneal injections of sulpiride (10 mg/kg) twice a week. Mice were euthanized following a 16‐week treatment period and a 1‐week behavioral test, after which brain and colon specimens were collected for qPCR analysis, western blot analysis, and histological evaluations.

### 4.4 Animal Experiment 3: Antibiotics‐Intervened Experiment

Male C57BL/6J mice (6 weeks old, SPF) were randomly assigned to four groups (*n* = 9 per group): (1) EtOH group: mice were fed an AIN93M ethanol liquid diet and orally gavaged daily with sterilized CMC‐Na (0.05%); (2) ABX‐EtOH group: mice were fed an AIN93M ethanol liquid diet with antibiotic treatment (twice/week) and orally gavaged daily with sterilized CMC‐Na (0.05%); (3) EtOH‐UA group: mice were fed an AIN93M ethanol liquid diet and orally gavaged daily with UA (200 mg/kg); (4) ABX‐EtOH‐UA group: mice were fed an AIN93M ethanol liquid diet and orally gavaged daily with UA (200 mg/kg) and antibiotic treatment (twice/week).

The pseudo‐germ‐free mice were treated with a broad‐spectrum antibiotic cocktail (comprising ampicillin, neomycin, and metronidazole at 1 g/L each; vancomycin at 0.5 g/L) for 14 days. Subsequently, the mice received weekly administrations of the same antibiotic cocktail until the end of the experiment to deplete the gut microbiota, consistent with the previously described [75]. The depletion of microbiota was confirmed through plating fecal samples on brain heart infusion (BHI) agar plates at 37°C under both anaerobic and aerobic conditions, as well as qRT‐PCR using the universal bacteria‐specific primer pair. The mice were euthanized following a 16‐week treatment period and a 1‐week behavioral test, after which brain and colon specimens were collected for qPCR analysis, western blot analysis, and histological evaluations.

### 4.5 Animal Experiment 4: FMT Experiment

Male C57BL/6J mice (6 weeks old, SPF) were randomly assigned to four groups (*n* = 10 per group): (1) Ctrl group: mice were fed an AIN93M control liquid diet and received daily oral gavage of fecal suspension from the Ctrl group; (2) Ctrl‐FMT**^d‐EtOH^** group: mice were fed an AIN93M control liquid diet and received daily oral gavage of fecal suspension from the EtOH group; (3) EtOH group: mice were fed an AIN93M ethanol liquid diet and received daily oral gavage of fecal suspension from the EtOH group; (4) EtOH‐FMT**^d‐EtOH‐UA^**: mice were fed an AIN93M ethanol liquid diet and received daily oral gavage of fecal suspension from the EtOH‐UA group;

Donor Mice were housed in a sterile plastic cage without bedding and sterilized with 75% ethanol until defecation occurred during fecal material collection of fecal material. To ensure an adequate volume of FMT material while avoiding potential cross‐contamination, the collection was staggered over six to nine days per group. Fecal pellets were promptly collected and snap‐frozen in liquid nitrogen for subsequent microbiome analysis, followed by storage at −80°C until sequencing. Freshly collected fecal specimens were immediately transferred to an anaerobic chamber for categorical pooling to achieve sufficient microbial biomass. For the FMT experiment, aliquots were prepared by homogenizing 1 g samples in 5 mL sterile PBS supplemented with 20% glycerol (w/v), followed by sequential filtration through 70‐µm meshes and cryopreservation at −80°C.

Recipient pseudo‐germ‐free mice were generated through a 14‐day antibiotic regimen administered via liquid diet, containing ampicillin, neomycin, and metronidazole (1 g/L each) with vancomycin (0.5 g/L). Prior to FMT administration, intestinal decontamination was performed using polyethylene glycol solution (PEG 4000, 59 g/L) containing electrolyte additives: NaCl (1.46 g/L), KCl (0.75 g/L), Na_2_SO_4_ (5.68 g/L), and NaHCO_3_ (1.68 g/L). Each mouse received 1.5 mL of the intestinal bowel cleansing solution via oral gavage, with two administrations of 500 µL on the day preceding FMT at a one‐hour interval, followed by an additional administration of 500 µL four hours before FMT. Mice were fasted for 4 h before PEG 4000 administration, and the bedding was replaced after each intestinal purge in consideration of their coprophagous behavior. FMT inoculum (100 mg/mL in sterile PBS) was delivered twice weekly via oral gavage (400 µL/dose) using sterilized gastric needles. To ensure proper microbiota colonization, a priming phase with daily administrations was implemented during the initial 7 days, followed by a maintenance regimen of biweekly dosing. The mice were euthanized following a 16‐week treatment period and a 1‐week behavioral test, after which brain and colon specimens were collected for qPCR analysis, western blot analysis, and histological evaluations.

### 4.6 Animal Experiment 5: *B. sartorii* and *P. distasonis* Treatment Experiment

Male C57BL/6J mice (6‐week‐old, SPF) were randomly assigned to four groups (*n* = 10 per group): (1) Ctrl group: mice were fed an AIN93M control liquid diet and received daily oral gavage of sterile PBS (200 µL/mouse) for 16 weeks; (2) EtOH group: mice were fed an AIN93M ethanol liquid diet with equivalent PBS administration for 16 weeks; (3) EtOH‐*B. sartorii* group: mice were fed an AIN93M ethanol liquid diet and received daily oral gavage of *B. sartorii* suspension (10^9 ^cfu/mL in sterile PBS) per mouse daily for 16 weeks; (4) EtOH‐*P. distasonis* group: mice were fed an AIN93M ethanol liquid diet and received daily oral gavage of *P. distasonis* suspension (10^9 ^cfu/mL in sterile PBS) per mouse daily for 16 weeks. The mice were euthanized following a 16‐week of treatment period and a 1‐week behavioral test, after which brain and colon specimens were collected for qPCR analysis, western blot analysis, and histological evaluations.

### 4.7 Animal Experiment 6: AEA Treatment Experiment

Male C57BL/6J mice (6‐week‐old, SPF) were randomly assigned to three groups (*n* = 10 per group): (1) Ctrl group: mice were fed an AIN93M control liquid diet, and treated with intraperitoneal injections of sterilized saline every two days; (2) EtOH group: mice were fed an AIN93M ethanol liquid diet, and treated with intraperitoneal injections of sterilized saline every two days; (3) EtOH‐AEA group: mice were fed an ethanol liquid diet with intraperitoneal injections of AEA at a dosage of 10 mg/kg twice a week. The mice were euthanized following a 16‐week treatment period and a 1‐week behavioral test, after which brain and colon specimens were collected for the evaluation of qPCR analysis, western blot analysis, and histological evaluations.

### 4.8 Animal Experiment 7: CB1R Inhibition Experiment

Male C57BL/6J mice (6‐week‐old, SPF) were randomly assigned to four groups (*n* = 10 per group): (1) Ctrl group: mice were fed an AIN93M control liquid diet and orally gavaged with sterilized CMC‐Na (0.05%), and treated with intraperitoneal injections of sterilized saline twice a week; (2) EtOH group: mice were fed an AIN93M ethanol liquid diet and orally gavaged with sterilized CMC‐Na (0.05%), and treated with intraperitoneal injections of sterilized saline twice a week; (3) EtOH‐UA group: mice were fed an AIN93M ethanol liquid diet and orally gavaged daily with UA (200 mg/kg/day). Additionally, they received intraperitoneal injections of sterilized saline twice a week; (4) EtOH‐UA‐AM251 group: mice were fed an AIN93M ethanol liquid diet and orally gavaged with UA (200 mg/kg/day), and treated with intraperitoneal injections of AM251 (3 mg/kg) at a dosage of 3 mg/kg twice a week. The mice were euthanized following a 16‐week treatment period and a 1‐week behavioral test, after which brain and colon specimens were collected for the evaluation of qPCR analysis, western blot analysis, and histological evaluations.

### 4.9 Animal Experiment 8: Delayed UA Treatment Experiment

Male C57BL/6J mice (6‐week‐old, SPF) were randomly assigned to four groups (*n* = 10 per group). (1) Ctrl group: mice were fed an AIN93M control liquid diet and orally gavaged with sterilized CMC‐Na (0.05%); (2) EtOH group: mice were fed an AIN93M ethanol liquid diet and orally gavaged with sterilized CMC‐Na (0.05%); (3) EtOH‐UA group: mice were fed an ethanol liquid diet and orally gavaged daily with UA (200 mg/kg/day) dissolved in sterilized CMC‐Na (0.05%). (4) EtOH‐UA‐Delayed group: mice were fed an ethanol liquid diet for 16 weeks, and orally gavaged daily with UA (200 mg/kg/day) dissolved in sterilized CMC‐Na (0.05%) from week 8 to week 16. The mice were euthanized following a 1‐week behavioral test, after which brain and colon specimens were collected for the evaluation of qPCR analysis, western blot analysis, and histological evaluations.

### 4.10 Animal Experiment 9: UA and Antibiotics‐Intervened Experiment on Control‐Diet Mice

Male C57BL/6J mice (6‐week‐old, SPF) were randomly assigned to four groups (*n* = 9 per group): (1) Ctrl group: mice were fed an AIN93M control liquid diet and orally gavaged with sterilized CMC‐Na (0.05%); (2) ABX‐Ctrl group: mice were fed an AIN93M control liquid diet with ABX treatment (twice/week) and daily orally gavaged with sterilized CMC‐Na (0.05%); (3) Ctrl‐UA group: mice were fed an AIN93M control liquid diet and orally gavaged daily with UA (200 mg/kg); (4) ABX‐Ctrl‐UA group: mice were received an AIN93M control liquid diet with ABX treatment (twice/week) and orally gavaged daily with UA (200 mg/kg). The mice were euthanized following a 16‐week treatment period and a 1‐week behavioral test, after which brain and colon specimens were collected for the evaluation of qPCR analysis, western blot analysis, and histological evaluations.

### 4.11 Y‐Maze

The Y‐maze experiment was conducted to assess spontaneous alternation behavior. Spontaneous alternation was defined as consecutive entries into the three arms in overlapping triplet sets. Each arm measured 35 cm in length, 15 cm in height, and 5 cm in width, converging at an equilateral angle (Shanghai Xin Ruan Information Technology, Shanghai, China). Mice were placed at the center of the maze and allowed to freely explore for 5 min. The total number of arm entries and alternations was recorded. Percent alternation was calculated as the ratio of actual alternations to possible alternations (defined as the total number of arm entries − 2) × 100%.

### 4.12 Novel Object Recognition

The novel object recognition test is a relatively low‐stress and efficient paradigm for evaluating short‐term learning and memory abilities in mice [79]. Considering the inherent inclination of mice toward novelty, if a mouse exhibits recognition of a familiar object, it will predominantly allocate time to exploring the novel object. The NOR paradigm employed a standardized three‐day protocol comprising habituation, training, and testing phases. In the habituation phase, individual mice were acclimated to a sterile open‐field arena (40 × 40 × 40 cm^3^) through 5‐min of unconstrained exploration, followed by temporary housing in a clean holding cage until all cohort members completed the session. During training trials, two isomorphic objects were positioned in symmetrical quadrants of the arena, with subjects initiated at the geometric center for 5‐min object interaction periods. The critical testing phase involved replacing one familiar object with a novel counterpart, allowing quantification of novelty preference through automated tracking of exploration time. The *discrimination ratio* = (*N* − *F*)/(*N* + *F*), where *N* and *F* denote time invested in novel and familiar objects, respectively.

### 4.13 Barnes Maze

Barnes maze is s a widely recognized classic paradigm for assessing the spatial learning and memory ability of mice [80]. The Barnes maze consisted of a circular, gray platform with a height of 140 cm (diameter = 122 cm). There were 19 equidistant circular holes (*d* = 5 cm) around the periphery of the platform. One of the holes was connected to a dark box, referred to as the target hole. The remaining round holes were voids that lack any connections. The target box was not visible from the platform surface, thereby rendering it inaccessible to animals. However, animals could still access the target box through the designated target hole. The experimental protocol consisted of a 5‐day localization cruise experiment followed by a 6‐day space exploration experiment. Each mouse was allotted a free exploration period of 5 min. The experimental protocol comprised a 5‐day localization cruise experiment, followed by a 6‐day space exploration experiment. Each mouse was allocated 5 min for free exploration.

### 4.14 Three‐Chamber Social Test

The three‐chamber sociability assay was conducted following an established protocol with three sequential phases [80]. After 5‐min habituation to a plexiglass arena (60 × 40 cm^2^) partitioned into three interconnected compartments, mice underwent sociability assessment through a binary choice paradigm. In the test phase, subjects were exposed simultaneously to an empty wire containment apparatus and an age/sex‐matched conspecific (Novel Mouse 1) in adjacent chambers. Social interaction parameters (olfactory investigation, climbing) were captured via automated tracking (Super Maze) and confirmed by blinded observers. The sociability index was computed as (T social – T empty)/(T social + T empty), where T represents time allocation. Social novelty preference was subsequently evaluated by replacing the empty container with a second novel conspecific (Novel Mouse 2), with the social novelty index calculated analogously as (T novel – T familiar)/(T novel + T familiar).

### 4.15 Transmission Electron Microscopy

Ultrastructural analysis of hippocampal CA1 synapses was conducted using a transmission electron microscope (TEM) (JEM‐1230, JEOL) following established histological processing protocols. Freshly dissected tissue underwent primary fixation in 2.5% glutaraldehyde (pH 7.2, 4°C, 24 h) followed by secondary fixation with 1% OsO_4_ (0.2 m PBS, pH 7.2, 4°C, 90 min). Stepwise dehydration was performed through a graded ethanol series (30%–100% v/v) before LR‐White resin infiltration (1:1 resin/ethanol overnight; pure resin 6 + 3 h). Polymerization at 60°C for 48 h preceded ultramicrotomy (Leica EM UC7), yielding 70 nm sections. Double‐contrast staining employed 3% uranyl acetate (10 min) and Reynold's lead citrate (8 min). Digital image acquisition utilized a Gatan 792 BioScan camera at 80 kV, with subsequent blinded morphometric analysis using Image J software (National Institutes of Health, Scion Corporation, USA) by experimenters who were blinded to the treatment groups.

### 4.16 Immunohistochemistry and Immunofluorescence Staining

Fixed cerebral sections underwent overnight incubation (4°C) with primary antibodies targeting either PSD‐95 or BDNF. Following three PBS rinses (5 min each), tissue specimens were exposed to biotin‐conjugated goat anti‐rabbit IgG (H+L) secondary antibodies (species‐specific, cross‐absorbed) at 37°C for 20 min. Post‐incubation washing involved six consecutive PBS cycles before DAPI counterstaining using antifade mounting medium. Fluorescent signals were captured through an Olympus IX83 inverted microscope (Tokyo, Japan) equipped with 3200‐series imaging components. Quantitative analysis of protein expression levels was conducted using ImageJ software, with fluorescence intensity normalized to background controls.

### 4.17 Fecal 16S rRNA Amplicon Sequencing and Microbiome Analysis

Fecal specimens were aseptically obtained from cohort‐matched mice during pre‐behavioral assessment phases and cryopreserved at −80°C prior to metagenomic analysis. Genomic DNA isolation was performed using the E.Z.N.A. Stool DNA Kit (Omega Bio‐tek, Norcross, GA) following standardized protocols. DNA integrity was verified through 1% agarose gel electrophoresis (120 V, 35 min), with quantification conducted via Qubit 4 Fluorometer (Thermo Fisher Scientific) using dsDNA BR Assay reagents. The hypervariable V3‐V4 regions were amplified using prokaryote‐specific primers (341F: 5’‐ACTCCTACGGGAGGCAGCAG‐3’; 806R: 5’‐GGACTACHVGGGTWTCTAAT‐3’) under thermocycling conditions optimized for Illumina platforms. Purified amplicons underwent paired‐end sequencing (2 × 300 bp) on HiSeq2500 (Illumina, San Diego, CA), yielding approximately 23,300 base‐pair reads per sample. Raw sequences were quality‐filtered (Q30 threshold) and merged into contigs using FLASH v1.2.11 (minimum overlap: 15 bp). Operational Taxonomic Units (OTUs) were clustered at 97% sequence similarity via USEARCH v7.0.1090, with chimera removal executed through UCHIME v4.2.40 against the Gold reference database. Taxonomic assignment was performed using the RDP Classifier v2.2 (confidence cutoff: 0.6) aligned to the Greengenes database (release 201305).

### 4.18 Principal Coordinate Analysis (PCoA) Based on Unweighted UniFrac Distances

Significant differences in microbial community structure between groups were tested by Permutational Multivariate Analysis of Variance (PERMANOVA) with 999 permutations, using the adonis2 function in the R package vegan. Beta diversity metrics (unweighted/weighted UniFrac distances) were computed in R v3.5.2 with subsequent PERMANOVA testing using the Adonis function from the Vegan package. Alpha diversity indices were compared between groups through nonparametric Wilcoxon rank‐sum tests. The correlation was calculated using R (v3.5.2) with Spearman's rank correlation coefficients, and subsequently adjusted for multiple comparisons using the *cor* test. Statistical significance was established at adjusted *p* < 0.05.

### 4.19 RNA Sequencing Analysis

Cryopreserved cerebral specimens were subjected to poly(A)+ RNA enrichment using oligo (dT)‐coupled magnetic beads (Thermo Fisher, MA, USA). RNA integrity was verified through Bioanalyzer 2100 (Agilent Technologies, CA, USA) prior to library construction. Fragmented RNA (median size: 250–300 nt) underwent first‐strand cDNA synthesis using SuperScript IV reverse transcriptase (Invitrogen) at 50°C for 15 min, followed by second‐strand synthesis with RNase H and DNA polymerase I (37°C, 1.5 h). Double‐stranded cDNA fragments were end‐repaired (T4 DNA polymerase, 20°C, 30 min), 3’‐adenylated (Klenow exo‐, 37°C, 30 min), and ligated to Illumina‐compatible adapters (T4 DNA ligase, 25°C, 15 min). Adapter‐ligated constructs were PCR‐amplified (12 cycles, KAPA HiFi HotStart) and circularized through splint ligation. Rolling circle amplification using Phi29 DNA polymerase generated DNA nanoballs (DNBs) that were subsequently arrayed on BGISEQ‐500 patterned nanochips (MGI Tech, Shenzhen, China). Sequencing was performed via combinatorial Probe‐Anchor Synthesis (cPAS) technology, generating 100 bp paired‐end reads. Raw reads were quality‐controlled using SOAPnuke (v2.3.5) with parameters: −Q 30 −n 0.01 −l 20. Processed reads were aligned to the UCSC mm10 reference genome using Bowtie2 (v2.4.2) in end‐to‐end mode. Transcript quantification was performed via RSEM (v1.3.1) with EBSeq‐adjusted expression values. DESeq2 (v1.4.5) implemented in the Dr. Tom Multi‐omics Platform (BGI‐Shenzhen) identified significantly dysregulated genes (FDR < 0.05, Wald test). Cluster visualization was achieved through pheatmap (v1.0.8) with Euclidean distance metric. Functional enrichment analysis of DEGs was conducted via hypergeometric testing using clusterProfiler (v3.18.1), with KEGG (https://www.kegg.jp/) and GO (http://www.geneontology.org/) annotations. *Q*‐values were used to correct for multiple testing, with a significance threshold of *Q* ≤ 0.05.

### 4.20 RNA Isolation and Real‐Time Quantitative PCR

Total RNA was isolated from mouse brain and colon tissues using the SteadyPure Universal RNA Extraction Kit II (Accurate Biotechnology, Hunan, China). RNA concentration and purity were assessed using a NanoDrop 2000 spectrophotometer (Thermo Scientific). Reverse transcription was performed with the Evo M‐MLV RT Mix Kit (Accurate Biotechnology) following manufacturer protocols, utilizing ≤100 ng total RNA in 20 µL reaction volumes. Quantitative PCR amplification was conducted using SYBR Green Premix Pro Taq HS qPCR Kit (Accurate Biotechnology, Hunan, China) on a CFX96 Touch Real‐Time PCR Detection System (Bio‐Rad). Gene‐specific primers (Sangon Biotechnology, Shanghai) are detailed in Table S2. The thermal cycling protocol comprised: initial denaturation at 95°C for 30 s, followed by 40 cycles of 95°C for 5 s and 60°C for 30 s. β‐actin served as the endogenous control for data normalization, with relative gene expression calculated using the 2−^△△^ *^Ct^* method.

### 4.21 Untargeted Metabolomics

Plasma samples were collected post‐euthanasia and stored at −80°C prior to analysis. Metabolite separation and detection were conducted using a Waters 2777C UPLC system coupled with a Q Exactive HF hybrid quadrupole‐Orbitrap mass spectrometer (Thermo Fisher Scientific). Chromatographic separation was achieved on an ACQUITY UPLC BEH C18 column (2.1 × 100 mm, 1.7 µm; Waters) maintained at 45°C. The mobile phase consisted of: (i) positive ion mode—0.1% formic acid (A) and acetonitrile (B); (ii) negative ion mode—10 mm ammonium formate (A) and acetonitrile (B). A gradient elution protocol was implemented: 0–1–min (2% B), 1–9 min (2%–98% B), 9–12 min (98% B), 12–12.1 min (98%–2% B), 12.1–15 min (2% B) at 0.35 mL/min flow rate with 5 µL injection volume. Mass spectrometric analysis employed the following parameters: Full‐scan range 70–1050 m/z at 120 000 resolution; AGC target 3 × 10^6^; maximum IT 100 ms. Top‐three precursors underwent MS/MS fragmentation (resolution 30 000; AGC 1 × 10^5^; max IT 50 ms) using stepped collision energies (20/40/60 eV). ESI settings: sheath gas 40 arb, aux gas 10 arb; spray voltage 3.80 kV (positive)/3.20 kV (negative); capillary temp 320°C; aux gas heater 350°C. Raw data were processed using Compound Discoverer 3.3 (Thermo Fisher Scientific) with reference to BMDB, mzCloud, and ChemSpider databases to generate a metabolite matrix containing peak areas and identification results. Subsequent bioinformatic processing included: data preprocessing and quality control, global metabolite profiling, differential analysis between comparison groups, and multi‐group comparative analysis.

### 4.22 Western Blot Analysis

Brain and colon tissue proteins (*n* = 3 biological replicates) were homogenized in lysis buffer and quantified. Protein lysates underwent SDS‐PAGE separation (10% resolving gel) followed by electrophoretic transfer to PVDF membranes using a Bio‐Rad Trans‐Blot system. Membranes were blocked with 5% non‐fat milk for 1 h at RT before sequential incubation with: (1) primary antibodies (4°C, overnight), (2) HRP‐conjugated secondary antibodies (RT, 1 h). Chemiluminescent signals were captured using ECL substrate and quantified via Quantity One software (Bio‐Rad). Band intensity normalization to β‐actin was performed using ImageJ (National Institutes of Health, Scion Corporation, USA).

### 4.23 Microbial Strain and Cultivation

All microbial strains (BeNa Biotechnology, BNCC) were maintained in an anaerobic chamber (Defender AMW1000, Hariolab) at 37°C. *Bacteroides sartorii* (JCM 17136) and *Parabacteroides distasonis* (DSM 20701) were respectively cultured on blood agar (HuanKai Microbial, 24070) and Columbia blood agar (LAND BRIDGE, PB003A) for 48 h under anaerobic conditions. Bacterial suspensions were prepared in sterile PBS to an OD600 = 1 ± 0.2 (mean ± SD) prior to oral administration.

### 4.24 Molecular Docking

Molecular docking simulations were conducted using CB‐DOCK2 [81, 82]. CB‐DOCK2 represented an advanced iteration of the protein–ligand blind docking software, incorporating both a curvature‐based cavity detection algorithm and a molecular docking protocol derived from AutoDock Vina. The workflow initiated with template ligand identification (FP2 similarity ≥ 0.4) from integrated protein–ligand databases. When template ligands were identified, the algorithm evaluated structural similarity between query proteins and template‐bound complexes, retaining only those meeting dual criteria: >40% sequence similarity and binding pocket RMSD ≤ 4 Å relative to templates. Final docking conformations were generated through FitDock's hierarchical alignment protocol, which optimizes initial template‐based poses followed by multi‐feature conformational exploration.

### 4.25 Quantification and Statistical Analysis

Statistical methods are detailed in the legends of each figure, with statistical significance established at *p* < 0.05 for mean comparisons. The number of animal replicates was determined based on power analysis derived from comparable prior studies. To retrospectively validate this sample size, we note that the key behavioral outcomes (e.g., Novel object recognition, Barnes maze, and Three‐chamber social test) yielded extremely significant differences (*p* < 0.001) with very large effect sizes (Cohen's *d* > 1.7), confirming the adequacy of our experimental design to detect robust effects. Data were analyzed using GraphPad Prism 9.0 (GraphPad Software Inc., San Diego, CA, USA), and all results except RNA sequencing and gut microbiome data are presented as mean ± SEM. Statistical comparisons between means were conducted using Student's *t‐*test or one‐way ANOVA as appropriate. For antibiotic treatment experiments, two‐way ANOVA was applied, considering UA and antibiotic treatment as independent variables. Post hoc analyses for multiple comparisons were performed using Tukey's test. For the correlation heatmap analysis, Spearman's rank‐order correlation was computed using the corr.test function from the R psych package, employing pairwise deletion to retain all valid data pairs. To mitigate false‐positive risks, the Holm correction method was implemented to control the family‐wise error rate, with statistical significance defined at α = 0.05. Additional software information is summarized in Table S1.

## Author Contributions

Conceptualization, M.W., Z.L. (Zhigang Liu), L.H., C.L., L.G., and H.Z.; Methodology, H.Z., Z.L. (Zibin Li), Y.X., L.G., and Z.L. (Zhigang Liu); Software, H.Z. and Z.L. (Zibin Li); Writing – Original Draft, H.Z.; Writing – Review & Editing, M.W., L.H., C.H., L.G., J.B., C.L., and H.Z.; Visualization, M.W., L.H., C.H., L.G., and H.Z.; Project Administration, M.W., L.H., L.G., and H.Z.; Supervision, M.W., L.H., C.H., L.G., and H.Z.; Funding Acquisition, M.W., Z.L. (Zhigang Liu), C.L., and L.H.

## Conflicts of Interest

The authors declare no conflict of interest.

## Supporting information




**Supporting File**: advs74164‐sup‐0001‐SuppMat.pdf.

## Data Availability

The raw data of RNA sequencing in the current study were deposited on GEO http://www.ncbi.nlm.nih.gov/geo/, and the accession number is GEO: GSE252138. The accession number for the entire 16S rRNA sequencing dataset reported in this manuscript is NCBI BioProject: PRJNA1057510 https://www.ncbi.nlm.nih.gov/bioproject/. Unprocessed data in this manuscript are available as Source Data in Supporting Information. Supplemental tables and figures are available as Supporting Information. Any additional information required to reanalyze the data reported in this work is available from the lead contact upon request.
