# Supplementary material for: Gut Microbiota‐Derived Anandamide Mediates the Therapeutic Effects of Urolithin A on Alcohol‐Induced Cognitive and Social Dysfunction via CB1R‐DRD2‐RAP1 Signaling Axis
Source: Adv Sci (Weinh). 2026 Feb 3;13(20):e08048. doi: 10.1002/advs.202508048 (PMC13067832; doi:10.1002/advs.202508048)
Supplement: Supplementary file 1 — Supporting File: advs74164‐sup‐0001‐SuppMat.pdf. [file ADVS-13-e08048-s001.pdf]

Gut Microbiota-Derived Anandamide Mediates the Therapeutic Effects of Urolithin A on Alcohol-Induced Cognitive and Social Dysfunction via CB1R-DRD2-RAP1 signaling Axis

Hongbo Zhang[1,2], Zibin Li[1], Yao Xiao[1], Ji Bian[3], Caian He [1], Chao Liu[5*], Lan Gong[4*], Lin Han[1*], Zhigang Liu [1*], and Min Wang[1*]*

[1] Department of Nutrition and Health, College of Food Science and Engineering, Northwest A&F University, Yang ling 712100, Shaanxi, China

[2] College of Food Science and Engineering, Ningxia University, Yin Chuan 750021, Ningxia, China

[3] Kolling Institute, Sydney Medical School, Royal North Shore Hospital, University of Sydney, St. Leonards, NSW 2065, Australia

[4] UNSW Microbiome Research Centre, St George and Sutherland Clinical Campus, University of New South Wales, Sydney, NSW 2052, Australia

[5] Key Laboratory of Novel Food Resources Processing, Ministry of Agriculture and Rural Affairs/Institute of Agro-Food Science and Technology, Shandong Academy of Agricultural Sciences, 202 Gongye North Road, Jinan 250100, P.R. China

Corresponding author:

Email: wangmin20050606@163.com (Min Wang)

Email: zhigangliu@nwsuaf.edu.cn (Zhigang Liu)

Email: hanlin730@163.com (Lin Han)

Email: l.gong@unsw.edu.au (Lan Gong)

Email: liuchao555@126.com (Chao Liu)

Email: zhanghongbo@nwafu.edu.cn (Hongbo Zhang)

Supplementary Table 1 Key Resources in Current Study

REAGENT or RESOURCE	SOURCE	IDENTIFIER
Antibodies		
β-actin	Abcam	Cat# ab8226; RRID:AB_306371
PSD95	Abcam	Cat# ab238135; RRID:AB_2895158
BDNF	Abcam	Cat# ab108319; RRID:AB_10862052
TNF-α	Abcam	Cat# ab183218; RRID:AB_2889388
APP	Abcam	Cat# 5382-1; RRID:AB_10896348
BACE1	Abcam	Cat# 2882-1; RRID:AB_2061494
IBA-1	Abcam	Cat# ab178847; RRID:AB_2832244
IL-10	Abcam	Cat# 1889-1; RRID:AB_764545
CB1R	Abcam	Cat# ab3558; RRID:AB_2229702
DRD2	Abcam	Cat# ab21218; RRID:AB_2277536
LPAR3	Thermo Fisher Scientific	Cat# PA5-27074; RRID:AB_2544550
RAP1	Abcam	Cat# 1726-1; RRID:AB_562208
Goat anti-Rabbit IgG (H+L) Cross-Adsorbed Secondary Antibody, HRP	Thermo Fisher Scientific	Cat# a16110; RRID:AB_2534783
Goat anti-Mouse IgG (H+L) Cross-Adsorbed Secondary Antibody, HRP	Thermo Fisher Scientific	Cat# a16078; RRID:AB_2534752
HRP-conjugated Affinipure Rabbit Anti-Goat IgG(H+L)	Proteintech	Cat# sa00001-4; RRID:AB_2864335
Bacterial and virus strains		
Bacteroids sartorii	JCM	JCM 17136
Parabacteroids distasonis	DSM	DSM 20701
Chemicals, peptides, and recombinant proteins		
Urolithin A	Nanjing DASF Biotechnology	CAS NO 1143-70-0
Metronidazole	Dalian Meilun biological Technology Co	CAS NO 443-48-1

Ampicillin	Dalian Meilun biological Technology Co	CAS NO 7177-48-2
Vancomycin hydrochloride	Dalian Meilun biological Technology Co	CAS NO 1404-93-9
Neomycin Sulfate	Dalian Meilun biological Technology Co	CAS NO 1405-10-3
Hematoxylin	Poly-scientific	S212
LR-White resin	London Resin Company	Cat# 14381-UC
Insulin	Solarbio	I8040
Fluorescein - Reference Standard	Thermo Fisher Scientific	F1300
Columbia blood agar plates	LAND BRIDGE	PB003A
Critical commercial assays		
AEA Kit	Baisimaike Bio.	BS-01459
SteadyPure Universal RNA Extraction Kit II	Accurate Biotechnology	Cat No. AG21022
cDNA using the Evo M-MLV RT Mix Kit with gDNA Clean for qPCR Ver.2	Accurate Biotechnology	Cat No. AG11728
SYBR® Green Premix Pro Taq HS qPCR Kit	Accurate Biotechnology	Cat No. AG11701
Deposited Data		
Raw and processed data (RNA-seq)	GEO (http://www.ncbi.nlm.nih.gov/geo/)	GEO: GSE252138
Raw and processed data (16S rRNA-seq)	NCBI BioProject (https://www.ncbi.nlm.nih.gov/bioproject/)	PRJNA1057510
Experimental models: Organisms/Strains		
Mice: C57BL/6J	Du Dossy Laboratory Animal Center	N/A
Oligonucleotides		
Primers for qPCR, See Table S2	Sangon Biotechnology	N/A
Software and algorithms		
ImageJ	National Institutes of Health	RRID: SCR_003070
Super Maze	XR-Xmaze	http://www.softmaze.com/
R (v4.2.1)	R Team	https://www.r-project.org/

GraphPad Prism 9.0	GraphPad Software	https://www.graphpad.com/
Bowtie2 (v2.2.5)	Langmead et al.[1]	http://bowtie-bio.sourceforge.net/bowtie2/index.shtml
RSEM (v1.2.8)	Li et al. [2]	http://deweylab.biostat.wisc.edu/rsem/rsem-calculate-expression.html
GO pathway database	N/A	https://geneontology.org/
KEGG pathway database	N/A	https://www.kegg.jp/
Phyper	N/A	https://en.wikipedia.org/wiki/Hypergeometric_distribution
pheatmap (v1.0.8)	N/A	https://cran.r-project.org/web/packages/pheatmap/
GSEA (v 4.0.3)	N/A	https://www.gsea-msigdb.org/gsea/index.jsp
Dr. Tom Multi-omics Data mining system	Bgi Genomics Co., Ltd	https://biosys.bgi.com
USEARCH (v7.0.1090)	N/A	http://www.drive5.com/usearch/
Fast Length Adjustment of Short reads, v1.2.11	N/A	https://mybiosoftware.com/flash-1-0-2-fast-length-adjustment-short-reads.html
Ribosomal Database Project Classifier (v2.2)	N/A	http://rdp.cme.msu.edu/
Other		
AIN93M - Sterile Control diet	TROPHIC Animal Feed High-tech Co.	TP 4020C
AIN93M - Sterile ethanol liquid diet	TROPHIC Animal Feed High-tech Co.	TP 4020A

Supplementary Table 2 List of Primers of qRT-PCR

Primers	Source	Identifier
Iba1-Seq (forward) 5′-TGACGGACCCCAAAAGATGA -3′	This paper	N/A
Iba1-Seq (reverse) 3′-TCTCCACAGCCACAATGAGT -5′	This paper	N/A
Drd2-Seq (forward) 5′-AGACACCACTCAAGGATGCTG -3′	This paper	N/A
Drd2 -Seq (reverse) 3′-TAGACCGTGGTGGGATGGAT -5′	This paper	N/A
Lpar3-Seq (forward) 5′-GACTGTGTCCAACCTCCTGG -3′	This paper	N/A
Lpar3 -Seq (reverse) 3′-CCACGAAGGCGCCTAAGAC -5′	This paper	N/A
H4c11 -Seq (forward) 5′-GGTGTTCCTGGAGAACGTGAT -3′	This paper	N/A
H4c11 -Seq (reverse) 3′-GGGAAATATAGTGTAAGCGAGCAG -5′	This paper	N/A
Loc100862468 -Seq (forward) 5′-ACCAAAGCAGCAGCCAATGAT -3′	This paper	N/A
Loc100862468 -Seq (reverse) 3′-GGCAAATGTATGAATGAAGACACCT -5′	This paper	N/A
Hnrnpa1l2-ps2 -Seq (forward) 5′-CTACCGTCATGTCTAAGTCCGAG -3′	This paper	N/A
Hnrnpa1l2-ps2 -Seq (reverse) 3′-CTCAGACTCTTGTCGGTTGTTTGA -5′	This paper	N/A
Zo1-Seq (forward) 5′-ACCCGAAACTGATGCTGTGGATAG -3′	This paper	N/A
Zo1-Seq (reverse) 3′-AAATGGCCGGGCAGAACTTGTGTA -5′	This paper	N/A
Occludin-Seq (forward) 5′-GGAGGACTGGGTCAGGGAATA -3′	This paper	N/A
Occludin-Seq (reverse) 3′-CGTCGTCTAGTTCTGCCTGT -5′	This paper	N/A
Muc1-Seq (forward) 5′-TCGTCTATTTCCTTGCCCTG -3′	This paper	N/A
Muc1-Seq (reverse) 3′-ATTACCTGCCGAAACCTCCT -5′	This paper	N/A
Muc2-Seq (forward) 5′-ACTGCACATTCTTCAGCTGC -3′	This paper	N/A
Muc2-Seq (reverse) 3′-ATTCATGAGGACGGTCTTGG -5′	This paper	N/A
Reg3y-Seq (forward) 5′-CAAGATGTCCTGAGGGC -3′	This paper	N/A
Reg3y-Seq (reverse) 3′-CCATCTTCACGTAGCAGC -5′	This paper	N/A

Reg3b-Seq (forward) 5′-GGCAACTTCACCTCACAT -3′	This paper	N/A
Reg3b -Seq (reverse) 3′-TGGGAATGGAGTAACAATG -5′	This paper	N/A
Fyb-Seq (forward) 5′-AAGTTGCAGGACAAAGCTCGCCT -3′	Adachi et al. [3]	N/A
Fyb-Seq (reverse) 3′-TCCTCGTAGGTAGGTTTCGCTGCC -5′	Adachi et al. [3]	N/A
Il1b-Seq (forward) 5′-GGTCAAAGGTTTGGAAGCAG -3′	Wang et al. [4]	N/A
Il1b-Seq (reverse) 3′-TGTGAAATGCCACCTTTTGA -5′	Wang et al. [4]	N/A
Tnfa-Seq (forward) 5′-CTCATGCACCACCATCAAGG -3′	Zhang et al. [5]	N/A
Tnfa-Seq (reverse) 3′-ACCTGACCACTCTCCCTTTG -5′	Zhang et al. [5]	N/A
Bdnf-Seq (forward) 5′-CTGGATGAGGACCAGAAG -3′	Sunkaria et al. [6]	N/A
Bdnf -Seq (reverse) 3′-CCTCCAGCAGAAAGAGTAG -5′	Sunkaria et al. [6]	N/A
Ngf-Seq (forward) 5′-CGACTCCAAACACTGGAACTCA -3′	Liu et al. [7]	N/A
Ngf -Seq (reverse) 3′-GCCTGCTTCTCATCTGTTGTCA -5′	Liu et al. [7]	N/A
Psd95-Seq (forward) 5′-TCTGTGCGAGAGGTAGCAGA -3′	Martin et al. [8]	N/A
Psd95-Seq (reverse) 3′-AAGCACTCCGTGAACTCCTG -5′	Martin et al. [8]	N/A
Fxr1-Seq (forward) 5′-GAGAGACTCGACATCAGCGA -3′	Guo et al. [9]	N/A
Fxr1-Seq (reverse) 3′-AGTGTCTGCAGTCTGATCGG -5′	Guo et al. [9]	N/A
Fxr2-Seq (forward) 5′-TCAAGACCCCAGAGACGAAA -3′	Guo et al. [9]	N/A
Fxr2-Seq (reverse) 3′-CTGAGGGTTTCGTGCGTTC -5′	Guo et al. [9]	N/A
Tdp2-Seq (forward) 5′-CCCATACTGTGCCTACCTAAAGA -3′	Liu et al. [10]	N/A
Tdp2-Seq (reverse) 3′-ACTCACATTTACGCATAGCAGG -5′	Liu et al. [10]	N/A
Glun2b-Seq (forward) 5′-AAGCCTGGCATGGTCTTCTC -3′	Kratsman et al. [11]	N/A
Glun2b -Seq (reverse) 3′-AGGTTGGCCATGTTTTTGGC -5′	Kratsman et al. [11]	N/A
Glua2-Seq (forward) 5′-AAAGAATACCCTGGAGCACAC -3′	Cook et al. [12]	N/A
Glua2 -Seq (reverse) 3′-CCAAACAATCTCCTGCATTTCC -5′	Cook et al. [12]	N/A

Gapdh-Seq (forward) 5′-TGGAGAAACCTGCCAAGTATGA -3′	Liu et al. [7]	N/A
Gapdh-Seq (reverse) 3′-TGGAAGAATGGGAGTTGCTGT-5′	Liu et al. [7]	N/A
The universal bacteria-Seq 8F: AGAGTTTGATCCTGGCTCAG	Xiao et al. [13]	N/A
The universal bacteria-Seq 338R: CTGCTGCCTCCCGTAGGAGT	Xiao et al. [13]	N/A

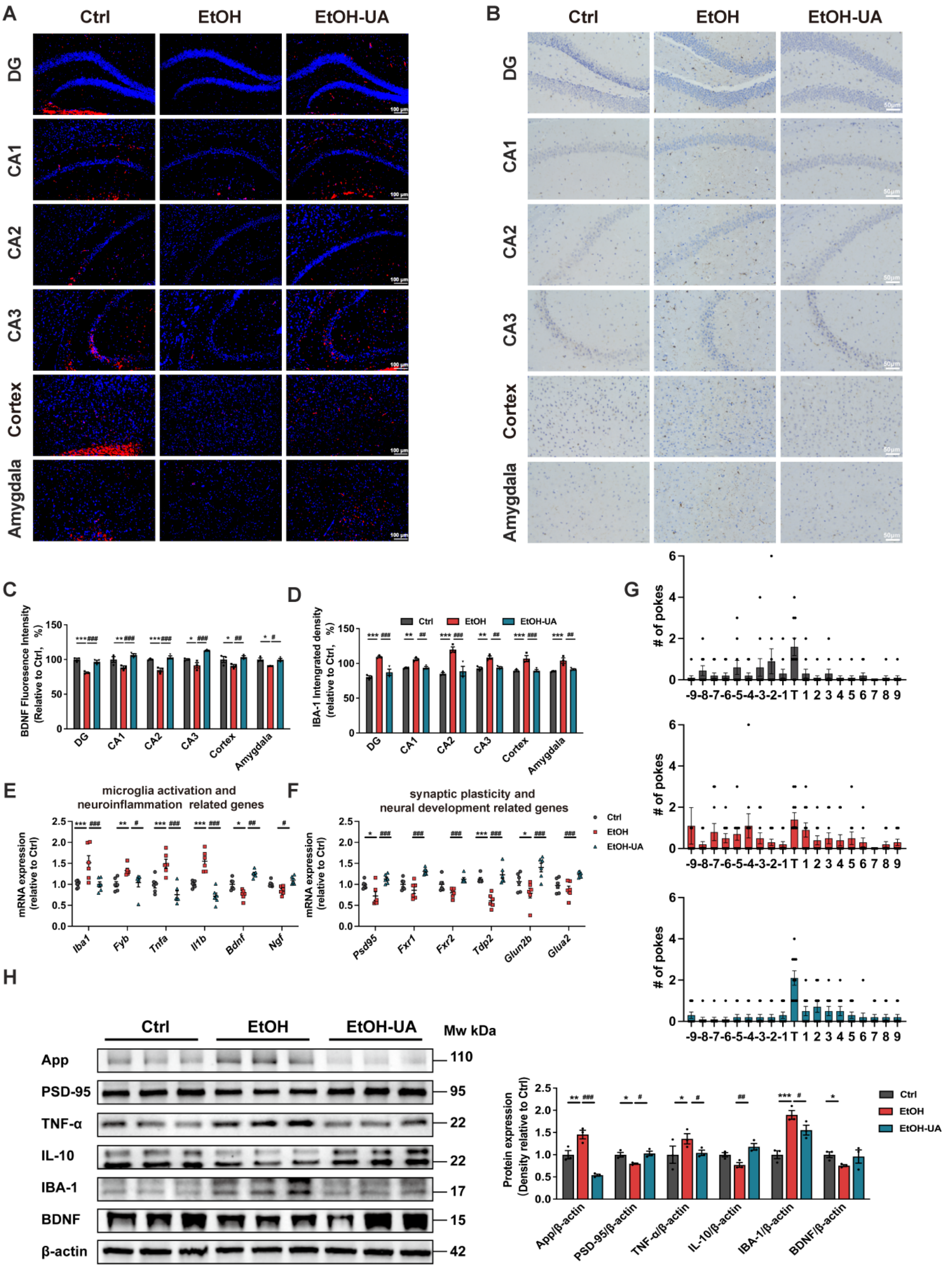

Supplementary Figure 1. UA ameliorates cognitive impairment, synaptic impairment, and neuroinflammation, related to Figures 1 and 2

(A) Representative images of immunofluorescence images of BDNF in the brain (*n* = 3 mice per group, scale bars, 100 μm).
(B) Immunochemical staining of IBA-1 of the brain (*n* = 3 mice per group, scale bars, 100 μm).
(C) Immunofluorescence intensity of BDNF.
(D) Immunochemical density of IBA-1.
(E) The relative mRNA levels of microglia activation and neuroinflammation in the hippocampus (n = 6 biologically independent samples per group).
(F) The relative mRNA levels of synaptic plasticity and neural development-related genes in the hippocampus (*n* = 6 biologically independent samples per group).
(G) Head exploring times in each hole in Barnes maze.
(H) Western blots analysis of synaptic plasticity and neuroinflammation-related proteins (*n* = 3 biologically independent samples per group).
Data presented as mean ± SEM. $*p < 0.05$, $**p < 0.01$, compared with Ctrl group, $^{\#}p < 0.05$, $^{\#\#}p < 0.01$ compared with the EtOH group. Significant differences between mean values were determined by one-way ANOVA with Tukey's multiple comparisons test.

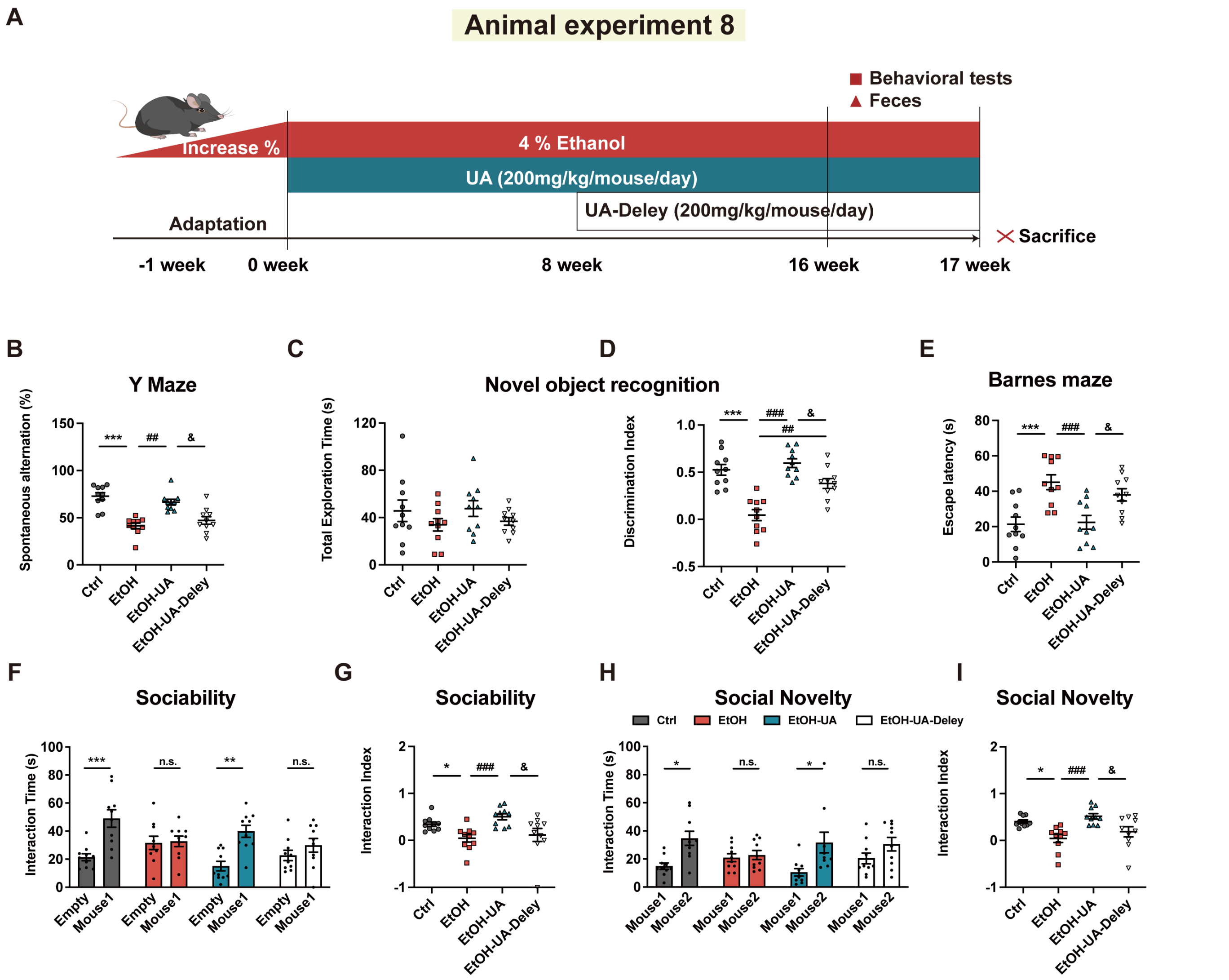

Supplementary Figure 2. Delayed administration of UA alleviates cognitive and social behavioral deficits, related to Figure 1

(A) Timeline of animal experiment 8 depicting the alcohol liquid diet of delayed UA treatment. (Ctrl group, EtOH group, and EtOH-UA group are the same in Figure 1)

(B) For the Y-maze, spontaneous alternations were recorded.

(C-D) For the novel object recognition test, the discrimination index between the novel and familiar objects was calculated.

(E) For the Barnes maze, escape latency was recorded.

(F&G) In the sociability test, the time spent interacting with a mouse or with an empty wire cage was recorded.

(H&I) In the social novelty test, the time spent interacting with a novel versus a familiar mouse was recorded.

Data presented as mean ± SEM. $^*p < 0.05$, $^{**}p < 0.01$, compared with Ctrl group, $^{\#}p < 0.05$, $^{\#\#}p < 0.01$ compared with the EtOH group, $^{\&}p < 0.05$, $^{\&\&}p < 0.01$ compared with the EtOH-UA group. Significant differences between mean values were determined by one-way ANOVA with Tukey's multiple comparisons test.

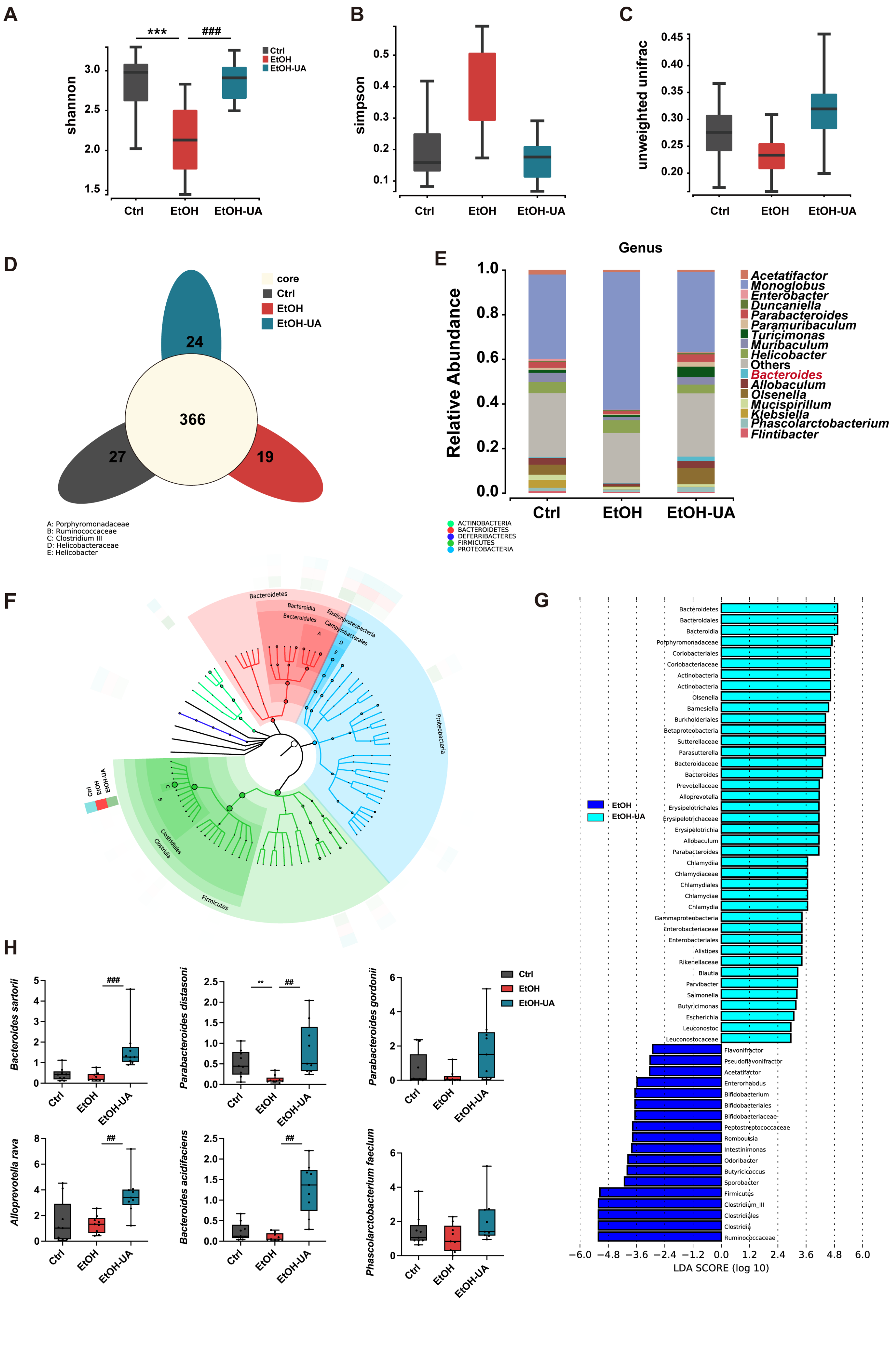

Supplementary Figure 3. UA restores alcohol-induced microbiota dysbiosis, related to Figure 3

(A) α diversity of Shannon.
(B) α diversity of Simpson.
(C) β diversity.
(D) OTU Core-Pan of OTUs were specific in different groups.
(E) The relative abundance of bacteria at the genus level. All genera with an average relative abundance below 0.5% were grouped to ‘‘others’’.
(F) GraPhlan species composition diagram showing the taxa most differentially associated with Ctrl (red), EtOH-UA (blue), and EtOH (green) (Wilcoxon rank-sum test). Circle sizes in the cladogram plot are proportional to bacterial abundance. The circles represent, going from the inner to outer circle: phyla, class, order, family, and genus.
(G) LEfSe / LDA Effect Size analysis of the bacteria at the genus level.
(H) Relative abundance of gut microbiota at the species level.
Data presented as mean ± SEM. $^{*}p < 0.05$, $^{**}p < 0.01$, compared with Ctrl group, $^{\#}p < 0.05$, $^{\#\#}p < 0.01$ compared with the EtOH group. Significant differences between mean values were determined by one-way ANOVA with Tukey’s multiple comparisons test.

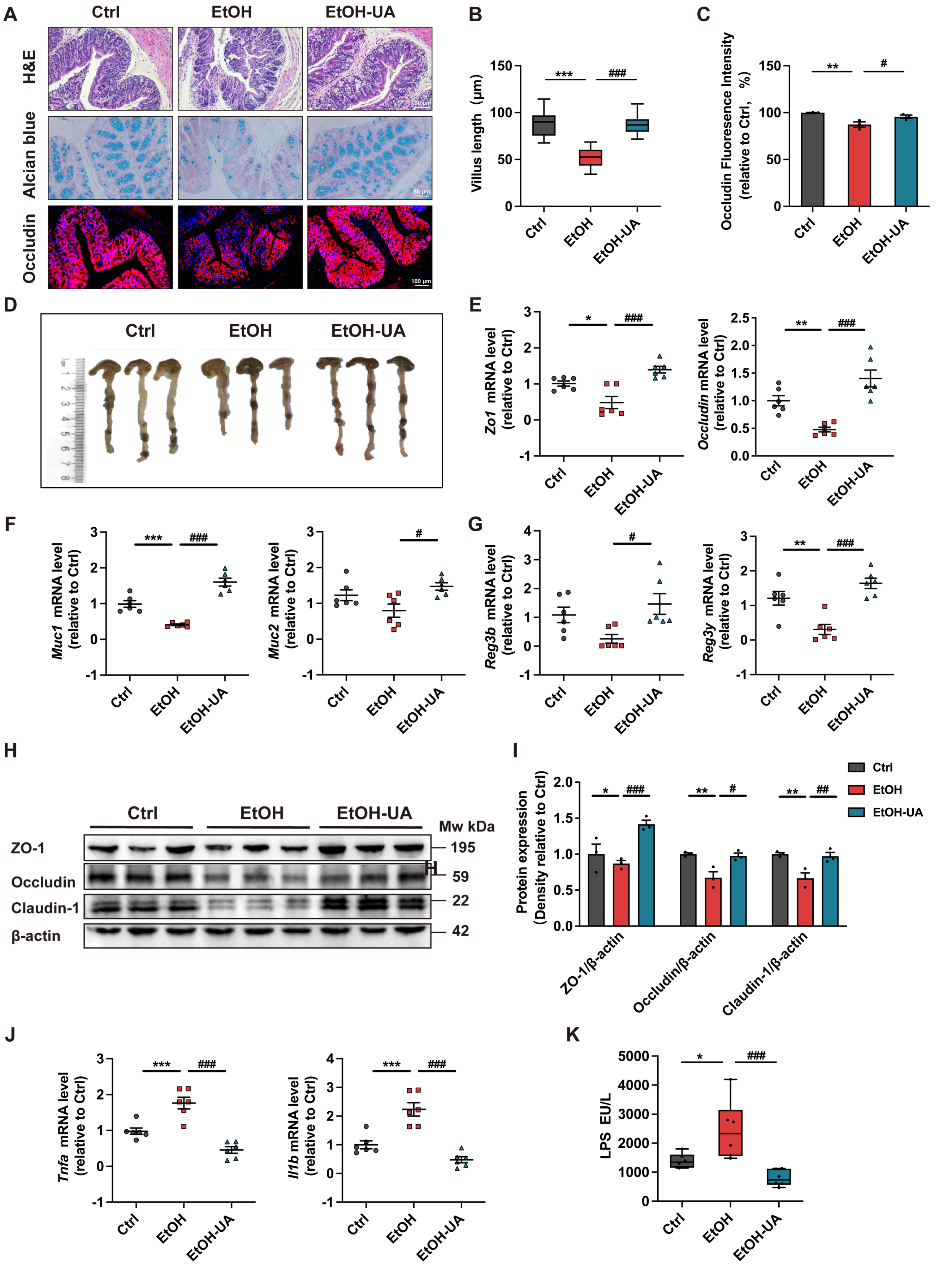

Supplementary Figure 4. UA restores alcohol-induced gut barrier impairment, related to Figure 3

(A) Representative images of H&E staining, alcian blue staining, and immunofluorescence images of Occludin in colon sections (n = 3 mice per group, scale bars, 50 μm).

(B) The villi length (n = 3 mice per group).

(C) Immunofluorescence intensity of Occludin.
(D) The length of the colon.
(E) The relative mRNA expressions of tight junction protein-related genes in the colon (*n* = 6 mice per group).
(F) The relative mRNA expressions of intestinal mucins genes in the colon (*n* = 6 mice per group).
(G) The relative mRNA expressions of intestinal antimicrobial peptides genes in the colon (*n* = 6 mice per group).
(H) Western blots analysis of tight junction-related proteins.
(I) Relative protein expression of tight junction-related proteins.
(J) The relative mRNA expressions of inflammation-related genes in the colon (*n* = 6 mice per group).
(K) Plasma LPS level.
Data presented as mean ± SEM. $^{*}p < 0.05$, $^{**}p < 0.01$, compared with Ctrl group, $^{\#}p < 0.05$, $^{\#\#}p < 0.01$ compared with the EtOH group. Significant differences between mean values were determined by one-way ANOVA with Tukey's multiple comparisons test.

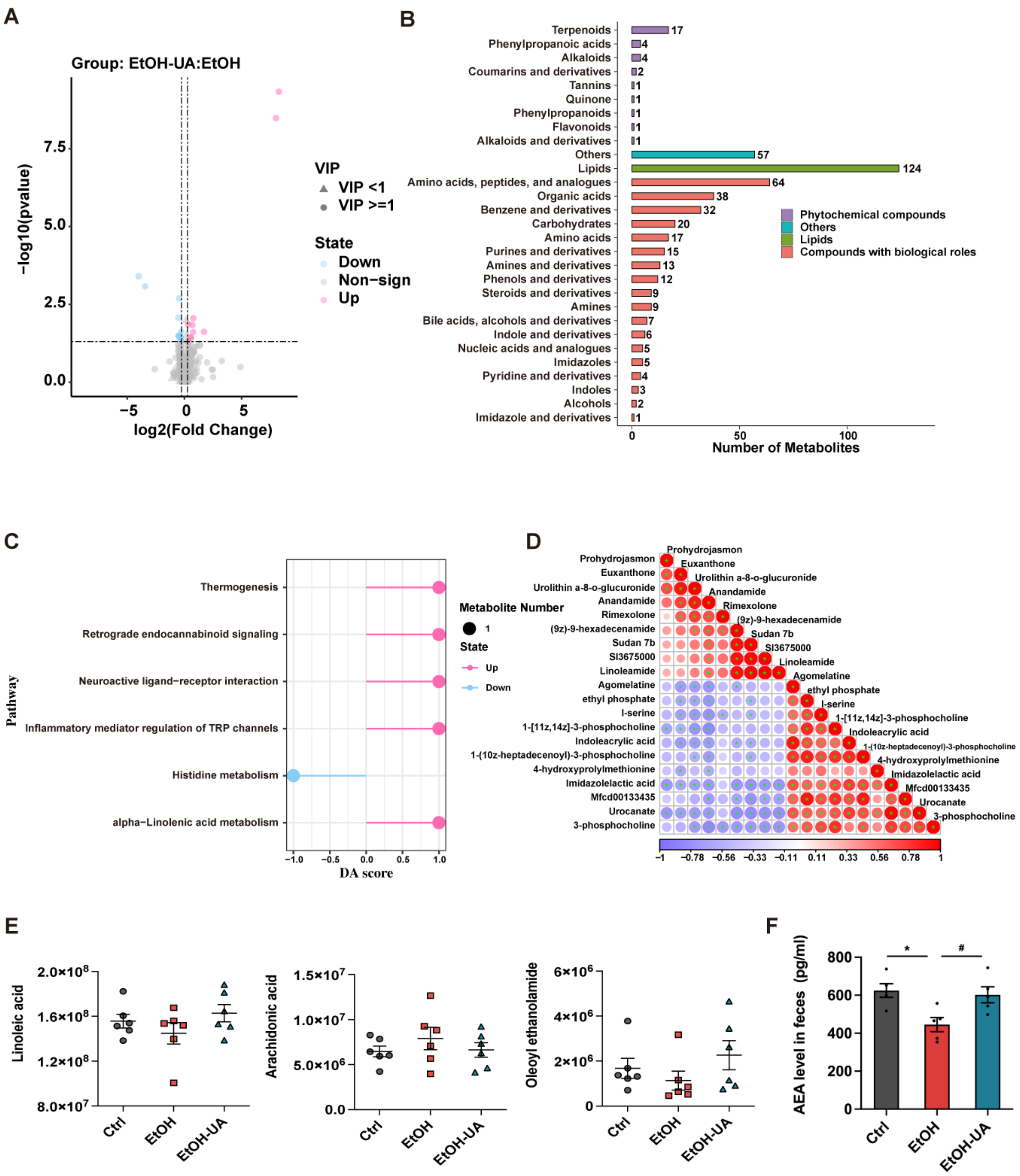

Supplementary Figure 5. The effects of UA on gut microbiota metabolites in chronic alcoholic mice, related to Figure 3

(A) Volcano plot based on the differential metabolites (EtOH-UA vs. EtOH).
(B) Metabolite Classification Bar Char.
(C) Correlation network diagram of differential metabolites.
(D) Metabolic pathway enrichment analysis abundance score plot.
(E) The relative levels of linoleic acid, arachidonic acid, and oleoyl ethanolamide.
(F) The concentration of AEA in feces (*n* = 5 mice per group).
Data presented as mean ± SEM. $^{*}p < 0.05$, $^{**}p < 0.01$, compared with Ctrl group, $^{\#}p < 0.05$, $^{\#\#}p < 0.01$ compared with the EtOH group. Significant differences between mean values were determined by one-way ANOVA with Tukey's multiple comparisons test.

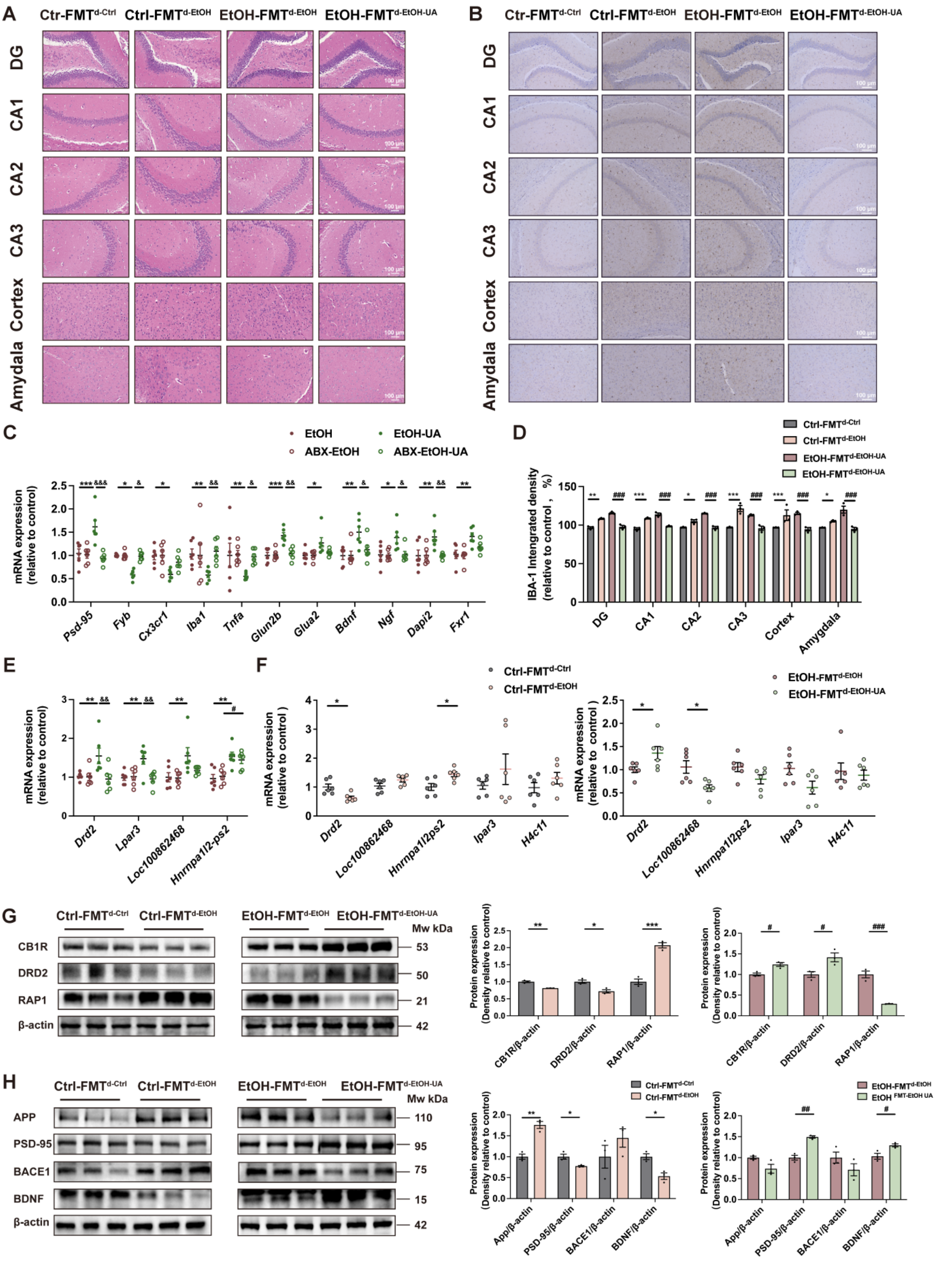

Supplementary Figure 6. UA restores synaptic impairments and neuroinflammation, and upregulates DRD2 in a gut microbiota-dependent manner, related to Figure 4

(A) Representative images of H&E staining of the brain (*n* = 3 mice per group, scale bars, 100 μm).
(B) Immunochemical staining of IBA-1 of the brain (*n* = 3 mice per group, scale bars, 100 μm).
(C) The relative mRNA levels of microglia activation, neuroinflammation, synaptic plasticity, and neural development-related genes in the hippocampus (*n* = 6 biologically independent samples per group).
(D) Immunochemical density of IBA-1.
(E) The relative mRNA levels of differential genes between EtOH and EtOH-UA group based on the RNA sequencing of the hippocampus in animal experiment 3 (*n* = 6 biologically independent samples per group).
(F) The relative mRNA levels of differential genes between EtOH and EtOH-UA group based on the RNA sequencing of the hippocampus in animal experiment 4 (*n* = 6 biologically independent samples per group).
(G) Western blots analysis of hippocampal CB1, DRD2, and RAP1 in animal experiment 4 (*n* = 3 biologically independent samples per group).
(H) Western blots analysis of cognitive-related proteins in animal experiment 4 (*n* = 3 biologically independent samples per group).
For the antibiotic experiment, data was presented as mean ± SEM. $^{*}p < 0.05$, $^{**}p < 0.01$, compared with EtOH group, $^{\#}p < 0.05$, $^{\#\#}p < 0.01$, compared with ABX-EtOH group, $^{\&}p < 0.05$, $^{\&\&}p < 0.01$ versus EtOH-UA group. Significant differences between mean values were determined by two-way ANOVA (UA and antibiotic treatment as two factors) with Tukey's multiple comparisons test.
For the FMT experiment, data was presented as mean ± SEM. $^{*}p < 0.05$, $^{**}p < 0.01$, compared with Ctrl-FMT$^{\mathbf{d\text{-}Ctrl}}$ group, $^{\#}p < 0.05$, $^{\#\#}p < 0.01$ compared with the EtOH-FMT$^{\mathbf{d\text{-}ETOH}}$ group. Significant differences between mean values were determined by one-way ANOVA with Tukey's multiple comparisons test.

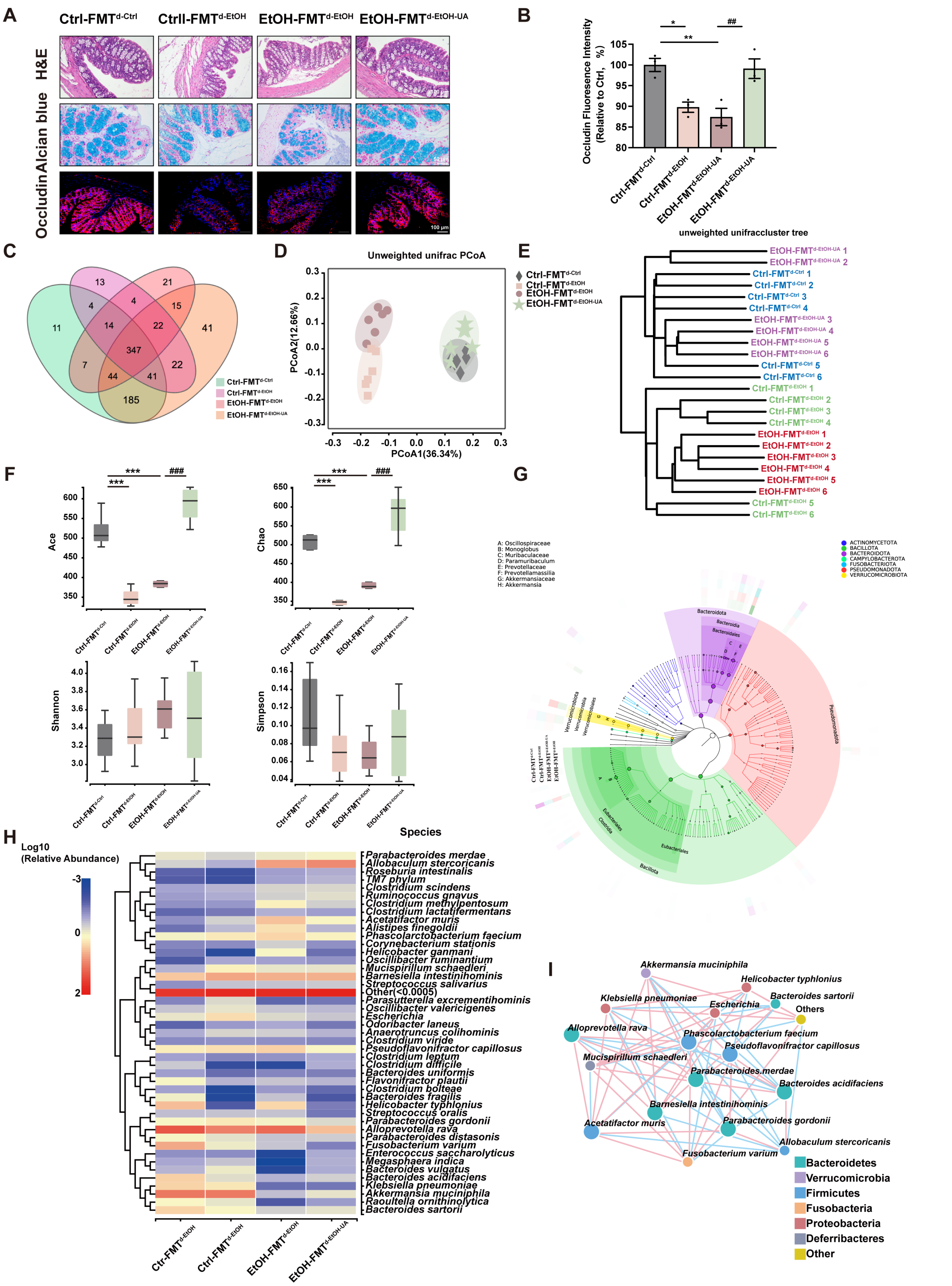

Supplementary Figure 7. The alterations in gut barrier and fecal microbial composition in the feces of mice with FMT treatment, related to Figure 4

(A) Representative images of HE staining (scale bars, 100 μm), alcian blue staining (scale bars, 50 μm), and immunofluorescence images of Occludin in colon sections (scale bars, 100 μm) ($n = 3$ mice per group). **(B)** The immunofluorescence intensity of Occludin.

(C) Venn diagram of OTUs shared among different groups.

(D) Principal coordinate analysis (PCoA) based on unweighted Unifrac distance and permutational manova (adonis) were used to test the difference in gut microbiota composition and diversity between groups**.**

(E) UPGMA cluster tree.

(F) α diversity of Ace, Chao, Shannon and Simpson.

(G) GraPhlan species composition diagram showing the taxa most differentially associated with Ctrl (red), EtOH-UA (blue), and EtOH (green) (Wilcoxon rank-sum test). Circle sizes in the cladogram plot are proportional to bacterial abundance. The circles represent, going from the inner to outer circle: phyla, class, order, family, and genus.

(H) Heatmap of the abundance of gut microbiota at the species level.

(I) Correlation network diagram of gut microbiota at the species level.

Data presented as mean ± SEM. $^{*}p < 0.05$, $^{**}p < 0.01$, compared with Ctrl-FMT$^{\mathbf{d\text{-}Ctrl}}$ group, $^{\#}p < 0.05$, $^{\#\#}p < 0.01$ compared with the EtOH-FMT$^{\mathbf{d\text{-}ETOH}}$ group. Significant differences between mean values were determined by one-way ANOVA with Tukey's multiple comparisons test.

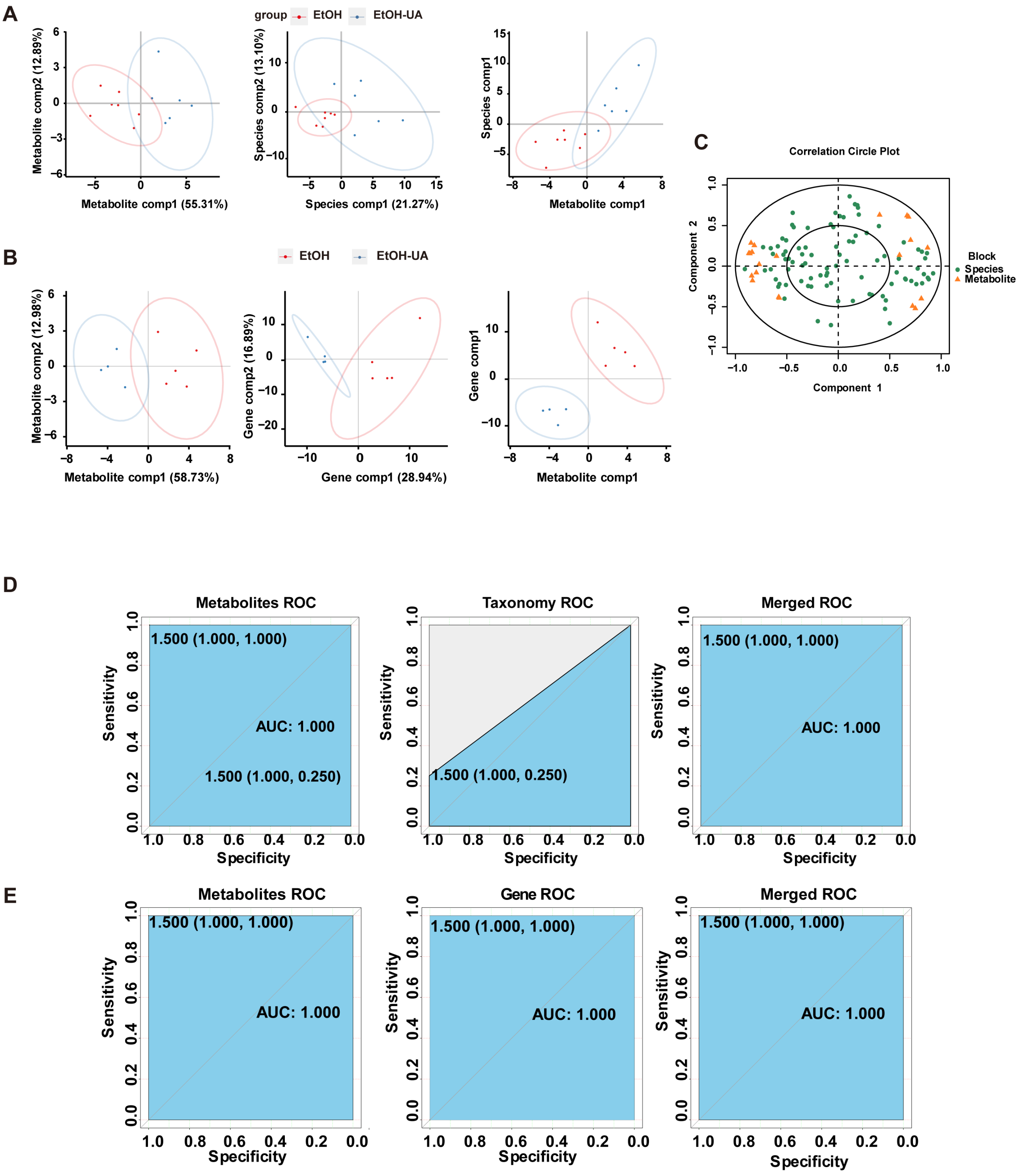

Supplementary Figure 8. Correlation of metabolomics with 16S rRNA and transcriptomics, related to Figures 5 and 7

(A) PCA map of microbial groups and metabolome (PCA map of metabolome is on the left, PCA map of microbial groups is in the middle, and PCA map of combined microbial groups and metabolome is on the right).

(B) PCA map of genes and metabolome (PCA map of metabolites is on the left, PCA map of genes is in the middle, and PCA map of combined genes and metabolites is on the right).

(C) Correlation Circle Plot for microbial groups and Differential Metabolites in the EtOH-UA group.

(D) The ROC curves of PLSDA analysis of microbial groups and metabolism biomarkers.

(E) The ROC curves of PLSDA analysis of genes and metabolism biomarkers.

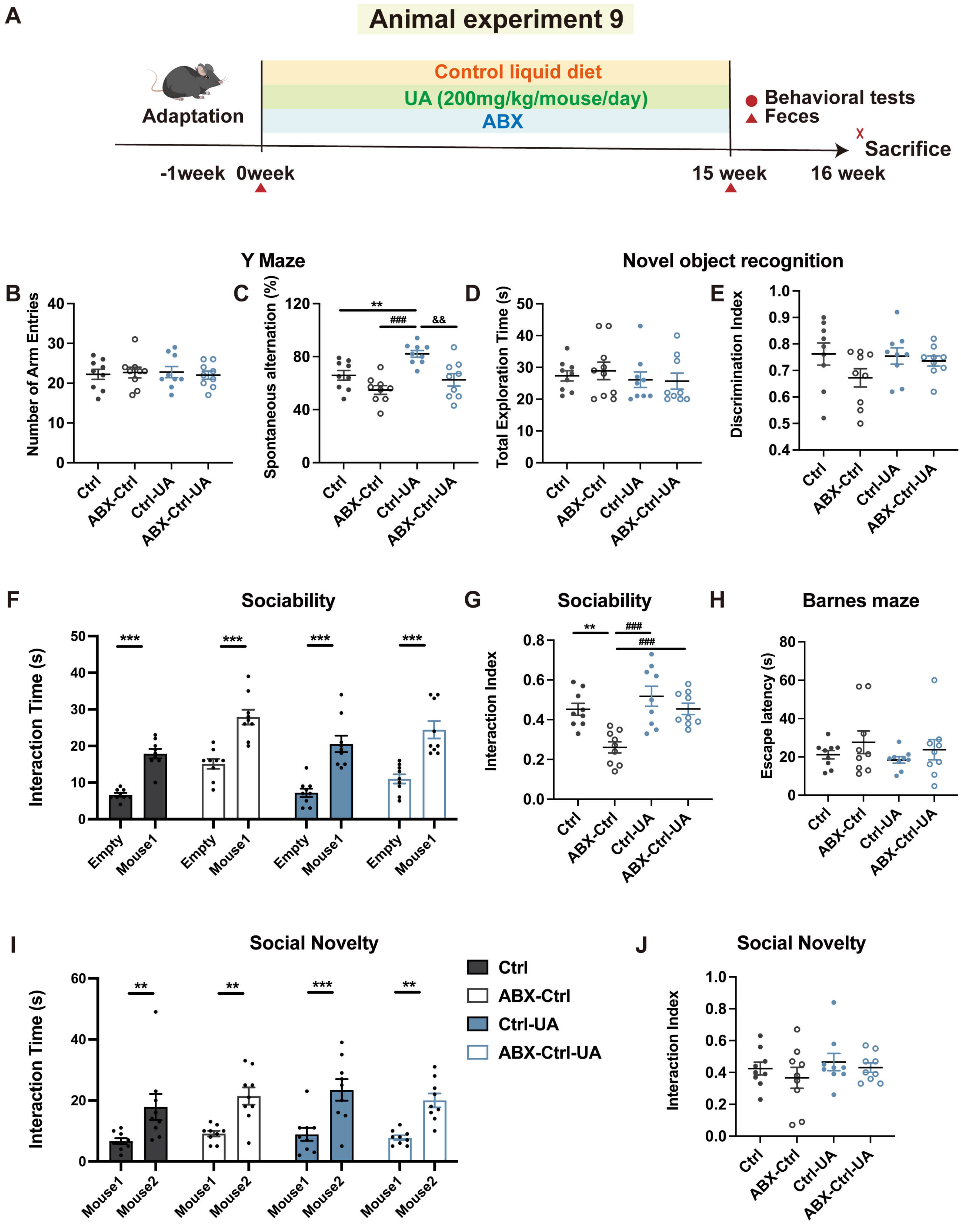

Supplementary Figure 9. Effects of UA and antibiotic treatment on cognitive and social functions in non-alcohol diet mice, related to Figure 4

(A) Timeline of animal experiment 9 depicting the non-alcohol diet with UA and antibiotic treatment.
(B-C) For the Y-maze, the number of arm entries and spontaneous alternations were recorded.
(D-E) For the novel object recognition test, the exploration time and discrimination index between the novel and familiar object were calculated.

(F-G) In the sociability test, the time spent interacting with a mouse or with an empty wire cage was recorded.
(H) For the Barnes maze, escape latency was recorded.
(I-J) In the social novelty test, the time spent interacting with a novel versus a familiar mouse was recorded. Data presented as mean ± SEM. $^{*}p < 0.05$, $^{**}p < 0.01$, compared with Ctrl group, $^{\#}p < 0.05$, $^{\#\#}p < 0.01$ compared with the ABX-Ctrl group, $^{\&}p < 0.05$, $^{\&\&}p < 0.01$ compared with the Ctrl-UA group. Significant differences between mean values were determined by two-way ANOVA (UA and antibiotic treatment as two factors) with Tukey's multiple comparisons test.

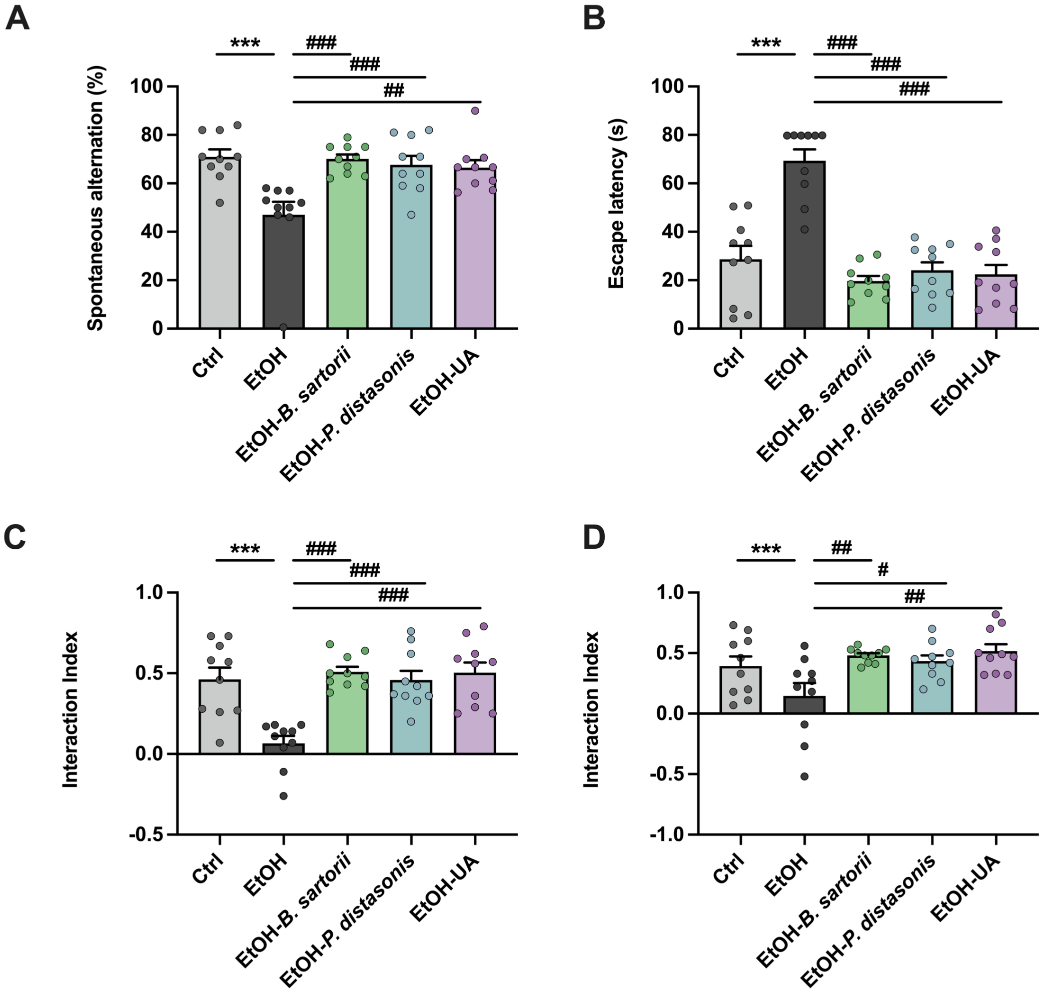

Supplementary Figure 10. Comparison of the therapeutic effects of UA treatment and monocolonization with *B. sartorii* or *P. distasonis* on AICSD-related behaviors.

(A) For the Y-maze, the number of arm entries and spontaneous alternations were recorded.

(B) For the Barnes maze, escape latency was recorded.

(C) In the sociability test, the time spent interacting with a mouse or with an empty wire cage was recorded.

(D) In the social novelty test, the time spent interacting with a novel versus a familiar mouse was recorded.

Data presented as mean ± SEM. *$p < 0.05$, **$p < 0.01$, compared with Ctrl group, $^{\#}p < 0.05$, $^{\#\#}p < 0.01$ compared with the EtOH group.

Supplementary References

1. Langmead, B. & Salzberg, S.L. Fast gapped-read alignment with Bowtie 2. *Nat. Methods* 2012; **9**: 357-U354.
2. Li, B. & Dewey, C.N. RSEM: accurate transcript quantification from RNA-Seq data with or without a reference genome. *BMC Bioinformatics* 2011; **12**: 323.
3. Adachi, M., Mizuno-Kamiya, M., Takayama, E., Kawaki, H., Inagaki, T., Sumi, S., *et al.* Gene expression analyses associated with malignant phenotypes of metastatic sub-clones derived from a mouse oral squamous cell carcinoma Sq-1979 cell line. *Oncol. Lett.* 2018; **15**: 3350-3356.
4. Wang, J., Zou, Q.H., Suo, Y., Tan, X.T., Yuan, T., Liu, Z.G., *et al.* Lycopene ameliorates systemic inflammation-induced synaptic dysfunction <i>via</i> improving insulin resistance and mitochondrial dysfunction in the liver-brain axis. *Food Funct.* 2019; **10**: 2125-2137.
5. Zhang, X., Zou, Q.H., Zhao, B.T., Zhang, J.W., Zhao, W.Y., Li, Y.T., *et al.* Effects of alternate-day fasting, time-restricted fasting and intermittent energy restriction DSS-induced on colitis and behavioral disorders *Redox Biol.* 2021; **44**: 101535.
6. Sunkaria, A., Yadav, A., Bhardwaj, S. & Sandhir, R. Postnatal Proteasome Inhibition Promotes Amyloid-β Aggregation in Hippocampus and Impairs Spatial Learning in Adult Mice. *Neuroscience* 2017; **367**: 47-59.
7. Liu, Q., Chen, Y.W., Shen, C., Xiao, Y.T., Wang, Y.T., Liu, Z.G., *et al.* Chicoric acid supplementation prevents systemic inflammation- induced memory impairment and amyloidogenesis via inhibition of NF-κB. *FASEB J.* 2017; **31**: 1494-1507.
8. Martin, V., Allaïli, N., Euvrard, M., Marday, T., Riffaud, A., Franc, B., *et al.* Effect of agomelatine on memory deficits and hippocampal gene expression induced by chronic social defeat stress in mice. *Sci. Rep.* 2017; **7**: 45907.
9. Guo, W.X., Polich, E.D., Su, J., Gao, Y., Christopher, D.M., Allan, A.M., *et al.* Fragile X Proteins FMRP and FXR2P Control Synaptic GluA1 Expression and Neuronal Maturation via Distinct Mechanisms. *Cell Rep.* 2015; **11**: 1651-1666.
10. Liu, X.N., Li, X., Xia, B., Jin, X., Zou, Q.H., Zeng, Z.H., *et al.* High-fiber diet mitigates maternal obesity-induced cognitive and social dysfunction in the offspring via gut-brain axis. *Cell Metab.* 2021; **33**: 923-938(e926).
11. Kratsman, N., Getselter, D. & Elliott, E. Sodium butyrate attenuates social behavior deficits and modifies the transcription of inhibitory/excitatory genes in the frontal cortex of an autism model. *Neuropharmacology* 2016; **102**: 136-145.
12. Cook, D., Nuro, E., Jones, E.V., Altimimi, H.F., Farmer, W.T., Gandin, V., *et al.* FXR1P Limits Long-Term Memory, Long-Lasting Synaptic Potentiation, and De Novo GluA2 Translation. *Cell Rep.* 2014; **9**: 1402-1416.
13. Xiao, X., Nakatsu, G., Jin, Y., Wong, S., Yu, J. & Lau, J.Y.W. Gut Microbiota Mediates Protection Against Enteropathy Induced by Indomethacin. *Sci. Rep.* 2017; **7**.
